# LncRNA IL21‐AS1 interacts with hnRNPU protein to promote IL21 overexpression and aberrant differentiation of Tfh cells in systemic lupus erythematosus

**DOI:** 10.1002/ctm2.1117

**Published:** 2022-11-29

**Authors:** Limin Liu, Longyuan Hu, Haojun Long, Meiling Zheng, Zhi Hu, Ye He, Xiaofei Gao, Pei Du, Hongjun Zhao, Di Yu, Qianjin Lu, Ming Zhao

**Affiliations:** ^1^ Department of Dermatology Hunan Key Laboratory of Medical Epigenomics, Second Xiangya Hospital, Central South University Changsha China; ^2^ Research Unit of Key Technologies of Diagnosis and Treatment for Immune‐related Skin Diseases Chinese Academy of Medical Sciences Changsha China; ^3^ Clinical Medical Research Center of Major Skin Diseases and Skin Health of Hunan Province Changsha China; ^4^ Department of Medical Science Laboratory The Fourth Affiliated Hospital of Guangxi Medical University Liuzhou China; ^5^ Department of Rheumatology Xiangya Hospital Central South University Changsha China; ^6^ The University of Queensland Diamantina Institute, Faculty of Medicine The University of Queensland Brisbane Queensland Australia; ^7^ Institute of Dermatology Chinese Academy of Medical Sciences and Peking Union Medical College Nanjing China

**Keywords:** autoimmunity, epigenetics, lncRNA, systemic lupus erythematosus

## Abstract

**Background:**

The aberrant differentiation of T follicular helper (Tfh) cells plays an important role in the pathogenesis of systemic lupus erythematosus (SLE). However, the mechanism of regulating Tfh cells differentiation remains unclear. Long noncoding RNAs (lncRNAs) act as important regulators in the processes of innate and adaptive immune response. Whether lncRNAs are involved in regulating Tfh cell differentiation and autoimmune responses need to be further identified.

**Methods:**

The characters and functions of human IL21‐AS1 and its mouse homologous lncRNA (mIl21‐AS) were investigated by a series of biochemical assays and cell transfection assay. mIl21‐AS1 regulating humoral immune response in vivo was explored by keyhole limpet haemocyanin (KLH) and chronic graft versus host disease (cGVHD) model.

**Results:**

Human IL21‐AS1 and its mouse homologous lncRNA (mIl21‐AS) were identified and cloned. We uncovered that IL21‐AS1 was highly expressed in CD4^+^ T cells of SLE patients and Tfh cells, which promoted differentiation of Tfh cells. Mechanistically, IL21‐AS1 bound heterogeneous nuclear ribonucleoprotein U and recruited acetyltransferases CREB‐binding protein to the promoter of *IL21*, leading to the transcriptional activation of *IL21* and Tfh cells differentiation through increasing Histone H3 acetylation level on *IL21* promoter. Moreover, Tfh proportion and antibodies production were significantly increased in mIl21‐AS knock‐in mice immunized with KLH. mIl21‐AS1 overexpression also exacerbated the lupus‐like phenotype in cGVHD mice model.

**Conclusions:**

Our results demonstrate that IL21‐AS1 activates *IL21* transcription via epigenetic mechanism to promote germinal centre response, adding insight into the molecular regulation of autoimmune pathogenesis and providing a novel target for SLE treatment.

## Graphical abstract

1. IL21‐AS1 was highly expressed in CD4^+^ T cells of SLE patients and Tfh cells specifically.
2. IL21‐AS1 binds to the promoter of *IL21* gene and interacts with hnRNPU and CBP protein to regulate H3 acetylation level in the promoter region of *IL21*.
3. IL21‐AS1 overexpression promotes *IL21* transcription activation and the aberrant differentiation of Tfh cells in SLE patients, thereby exacerbating autoimmune phenotypes of SLE.

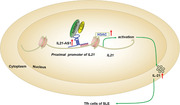

## 1 INTRODUCTION

Systemic lupus erythematosus (SLE) is a complex autoimmune disease, and the underlying aetiology and pathogenesis of SLE remain unclear.^1^ In general, genetic factor, environmental factors, epigenetic alteration, and response of immune system are involved in the pathogenesis of SLE.^2^, ^3^ Emerging research had reported the association between abnormal immune response and SLE.^4^ The immune disorders mainly include antigen presentation of dendritic cells (DC), inflammation promotion of helper T cell 1 (Th1) and Th17, and numerous autoantibodies secretion by B cells with the help of follicular helper (Tfh) cells.^2^

Tfh cells belong to CD4^+^ T cells that express CXCR5, inducible T‐cell co‐stimulator (ICOS), PD1, BCL6 and IL21.^5^, ^6^ Tfh cell regulates humoral immune response by assisting activation and differentiation of B cells to produce antibodies and contribute to the pathogenesis of SLE.^7^, ^8^ However, the mechanism that regulates Tfh cell activation and differentiation remains unclear. IL21, mainly secreted by Tfh and Th17 cells, plays a crucial role in promoting the differentiation of Tfh and Th17 cells, balancing subsets of helper T cells (Th), and the maturation and differentiation of B cells.^9^, ^10^ The mechanism that IL21 is overexpressed in SLE still requires to be further investigated.

Long noncoding RNAs (LncRNAs), commonly recognized as noncoding transcripts more than 200 nucleotides in length, are related with chromatin modification, transcriptional and post‐transcriptional regulation of genes.^11^, ^12^ LncRNAs participated in regulating the development, activation and differentiation of immune cells.^13^ Anti‐sense lncRNAs are at least partially complementary with protein‐coding transcripts, which may regulate the transcription of adjacent genes by modulating histone modifications.^14^ IL21 anti‐sense RNA 1 (IL21‐AS1) is a lncRNA that locates at the anti‐sense strand of *IL21* gene locus; however, the role and regulatory mechanism of IL21‐AS1 have not yet been reported.

In this study, we detected the expression of IL21‐AS1 in CD4^+^ T cells of SLE patients and lupus‐like mice model, cloned the full sequence of IL21‐AS1 and mouse Il21‐AS1 (mIl21‐AS1) and identified the intracellular localization of IL21‐AS1/mIl21‐AS1. We found that IL21‐AS1 overexpression promoted IL21 expression and Tfh cell differentiation. Moreover, we demonstrated that IL21‐AS1 regulated IL21 expression via binding heterogeneous nuclear ribonucleoprotein U (hnRNPU) and CREB‐binding protein (CBP) to increasing histone H3 acetylation (H3ac) modification at the promoter region of *IL21*. Our findings identified the characters of IL21‐AS1 and demonstrated the role and mechanism of IL21‐AS1 in regulating the aberrant Tfh cell differentiation in SLE.

## 2 RESULTS

### 2.1 IL21‐AS1 expression in CD4^+^ T cells of SLE patients and in vitro induced Tfh cells

**FIGURE 1:**
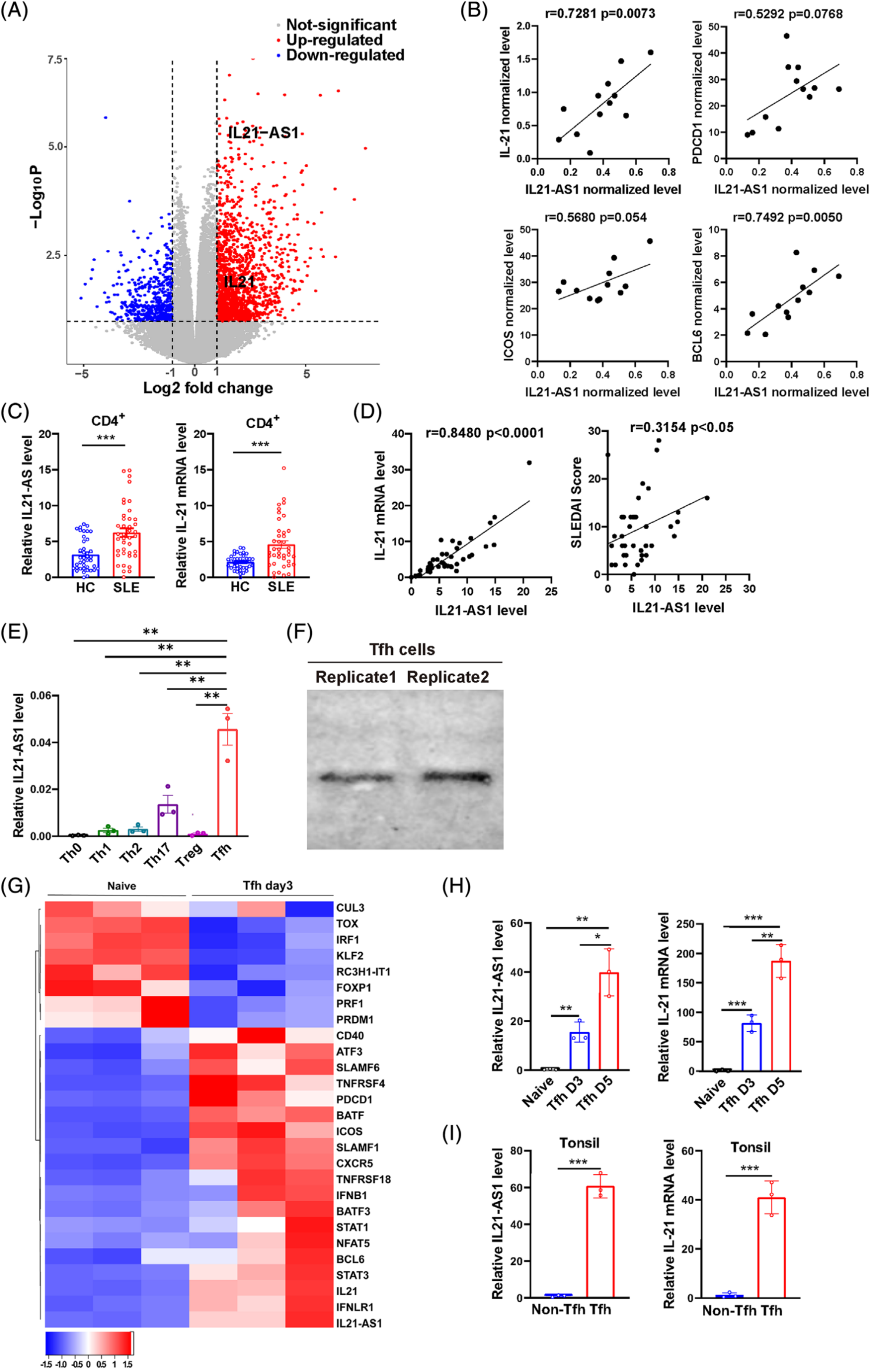
The expression of IL21‐AS1 in systemic lupus erythematosus (SLE) patients and Tfh cells. (A) Volcano map analysis of differential genes and long noncoding RNAs (lncRNAs) expression in CD4^+^ T cells of SLE patients (*n* = 12) and healthy controls (HCs, *n* = 8) by RNA‐seq. Red indicates up‐regulated genes and lncRNAs, and blue indicates down‐regulated genes and lncRNAs (log_2_ fold change ≥1 or ≤−1, *p*‐value <.05). (B) The correlation analysis between the expression of IL21‐AS1 and the expression of IL21, PDCD1, inducible T‐cell co‐stimulator (ICOS) and BCL6 in SLE patients according to RNA‐seq data (*n* = 12). (C) The expression levels of IL21‐AS1 and IL21 were significantly higher in CD4^+^ T cells of SLE patients compared with HCs (*n* = 40). (D) The correlation analysis between IL21‐AS1 expression level and IL21 mRNA level and SLEDAI score (*n* = 40). (E) RT‐qPCR detected the IL21‐AS1 expression levels in induced Th0, Th1, Th2, Th17 and Tfh cell under polarization condition in vitro. (F) Northern blot detected IL21‐AS1 expression in human‐Tfh5 cells. (G) RNA‐seq analysis of Tfh‐related genes expression between naïve CD4^+^ T cells and induced Tfh cells on day 3. (H) RT‐qPCR detection showed that the expression of IL21 and IL21‐AS1 was increased in the process of Tfh differentiation. (I) RT‐qPCR was used to detect the expression of IL21‐AS1 and IL21 mRNA in Tfh cells and non‐Tfh cells of Tonsil tissues. Data are representative of three independent experiments (mean ± SEM, *n* = 3). *p < .05, **p < .01, ***p < .001 relative to controls. *p*‐Value was determined using two‐tailed Student's *t*‐tests. Pearson correlation coefficient analysis was used in (B and D).

To screen the differently expressed lncRNAs in CD4^+^ T cells in SLE patients, we performed RNA‐seq in CD4^+^ T cells of 12 SLE and 8 healthy controls (HCs). We identified 2052 differentially expressed lncRNAs and genes (Table S1), among which IL21‐AS1 (NONCODE V5.0 database: NONHSAT098167.2) was up‐regulated in CD4^+^ T cells of SLE patients compared with HCs (Figure 1A). Moreover, we found a positive correlation between IL21‐AS1 level and IL21 and BCL6, respectively, especially IL21 expression in CD4^+^ T cells of SLE patients (Figure 1B). Furthermore, we verified the expression changes of IL21‐AS1 and IL21 by RT‐qPCR in CD4^+^ T cells of 40 SLE patients and 40 HCs. The results confirmed that in comparison with healthy donors, the expression levels of IL21‐AS1 and IL21 were notably increased in CD4^+^ T cells of SLE patients (Figure 1C), which was also positively correlated with IL21 transcription level and SLEDAI scores of SLE patients, suggesting IL21‐AS1 may be associated with the development of SLE (Figure 1D). Moreover, we observed no significant difference of IL21‐AS1 expression in CD19^+^ B cells and CD14^+^ monocytes of SLE patients compared with HCs, which indicated the cell specificity of IL21‐AS1 expression in SLE (Figure S1A,B). Moreover, we also detected the expression of IL21‐AS1 and IL21 in CD4^+^ T cells of systemic sclerosis, rheumatoid arthritis (RA) and psoriasis (PSO). The results showed that in comparison with HCs, IL21‐AS1 and IL21 expression were obviously up‐regulated in CD4^+^ T cells of RA patients (Figure S1C,D).

We also compared the expression level of IL21‐AS1 among different effector T cell subsets, which were induced from naïve CD4^+^ T cells in vitro. We found that IL21‐AS1 expression level was higher in Tfh cells than that in other cell subsets (Figure 1E). Northern blot also identified the mRNA expression of IL21‐AS1 in Tfh cells (Figure 1F). As IL21 is mainly secreted by Tfh cells, we induced Tfh cells differentiation under Tfh‐polarization culture condition and collected cells on day 3 and 5 to detect IL21‐AS1 expression. The results demonstrated that the mRNA levels of IL21‐AS1 and IL21 were notably up‐regulated on day 3 and 5 of Tfh differentiation compared to naïve CD4^+^ T cells (Figure 1G,H, Table S2). In addition, we detected IL21‐AS1 and IL21 expression in the CD4^+^PD‐1^+^CXCR5^+^ T (Tfh) cells and CD4^+^PD‐1^−^CXCR5^−^ T (non‐Tfh) cells from tonsil tissue from patients with suppurative tonsillitis. The result showed that in comparison with non‐Tfh cells, the levels of IL21‐AS1 and IL21 were obviously elevated in Tfh cells (Figure 1I).

### 2.2 Clone, identification and characterization of human IL21‐AS1 and its mouse homologous lncRNA

**FIGURE 2:**
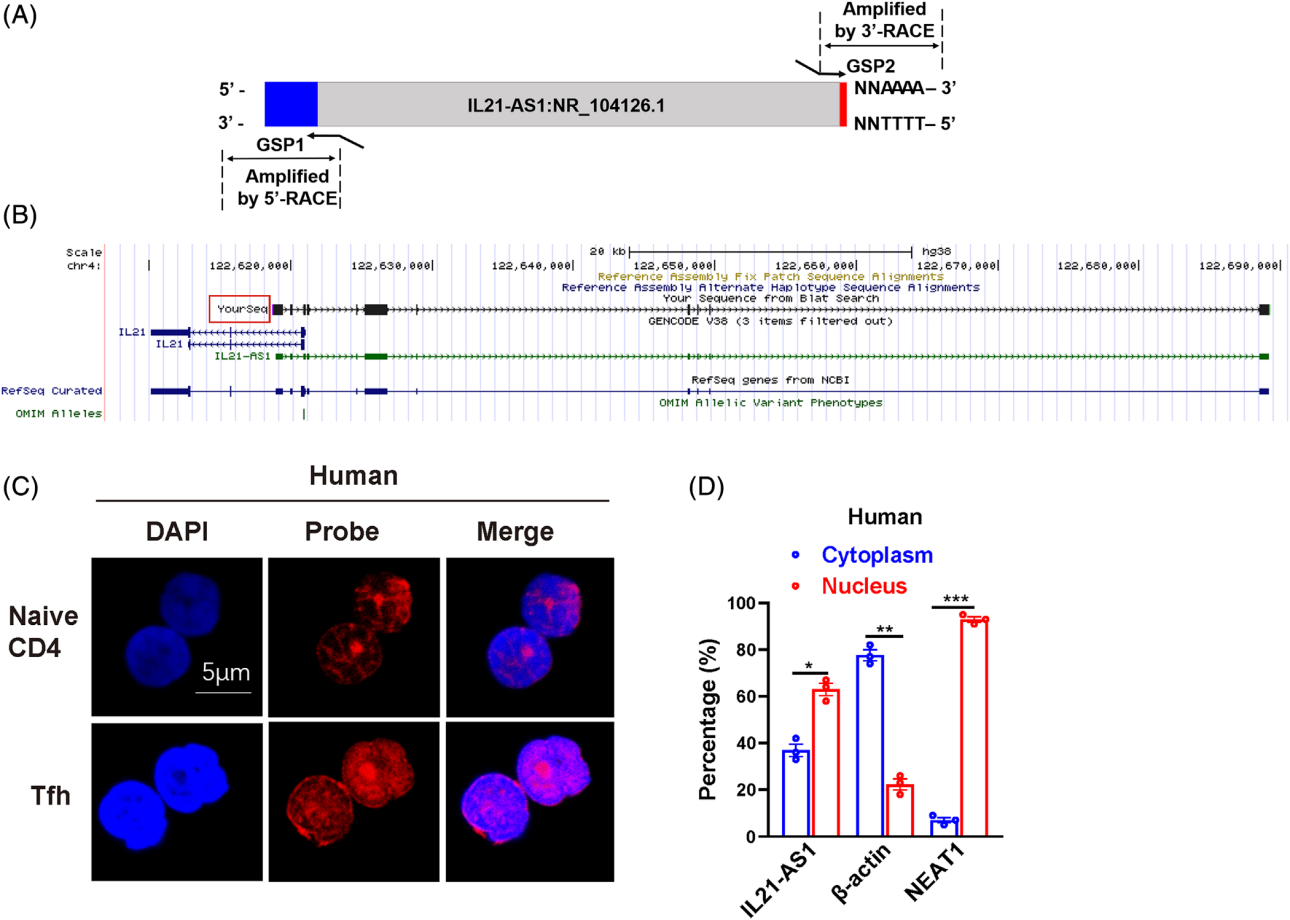
Cloning and identification of human IL21‐AS1. (A) The rapid amplifications of cDNA ends (RACE) amplification model of IL21‐AS1. (B) Sequence blast showed that the location of IL21‐AS1 was at the 5′ end of IL21 gene in the genome, opposite to transcription direction of IL21 gene. (C) Expression and distribution of IL21‐AS1 in human naïve CD4^+^ T cells and Tfh cells detected by FISH. Scale bar: 5 μm (D) RT‐qPCR identified the percentages of IL21‐AS1 expression in nucleus and cytoplasm of Tfh cells. Data are representative of three independent experiments (mean ± SEM, *n* = 3). *p < .05, **p < .01, ***p < .001 relative to controls. *p*‐Value was determined using two‐tailed Student's *t*‐tests.

Although human IL21‐AS1 has been identified as NONHSAG038804.2 with 3754 bp (NONHSAT098167.2) in noncoding RNA database, the full‐length sequence of IL21‐AS1 has not been confirmed. Here we performed the rapid amplifications of cDNA ends (RACE) to clone the 5′ and 3′ end of IL21‐AS1 sequence. According to the sequencing results of PCR production, the RNA full‐length sequence of IL21‐AS1 was determined to be 4111 bp with poly(A) tail after 321 and 36 bp extension to 5′ and 3′ terminals of IL21‐AS1 sequence, respectively (Figures 2A and S2A,B). In addition, we showed the genomic location of IL21‐AS1 RNA sequence (chr4:123,539,868‐123,610,322) with 11 exons, among which exons 3 and 4 are the anti‐sense sequence of 5′ end upstream of *IL21* gene (Figure 2B).

To identify the mouse homologous sequence of human IL21‐AS1, we first searched the transcripts located in the anti‐sense strand of *Il21* gene. Naïve CD4^+^ T cells from mouse spleen were isolated and polarized to Tfh cells in vitro. A transcript Gm12534, located in the anti‐sense of *Il21* gene, was identified in mouse polarized Tfh cells, and Gm12534 expression level was increased in polarized Tfh cells compared with naïve CD4^+^ T cells (Figure S3A,B). We cloned the full‐length sequence of the transcript to be 3181 bp length with poly(A) tail by 5′ and 3′‐RACE and named it after mIl21‐AS1 (Figures S2C,D and S3C,D). The mRNA of mIl21‐AS1 was also verified in the induced mouse Tfh cells by northern blot (Figure S3E).

Furthermore, to identify the intracellular localization of IL21‐AS1 and mIl21‐AS1 mRNA, we performed the RNA‐FISH in Tfh cells and naïve CD4^+^ T cells and RT‐qPCR detection using RNA from the separated nucleus and cytoplasm of Tfh cells. The results of FISH and RT‐qPCR showed that IL21‐AS1 and mIl21‐AS1 were expressed in both nucleus and cytoplasm, with more expression in nucleus (Figures 2C,D and S3F,G), and 18S RNA was used as cytoplasmic‐only control in FISH experiment (Figure S4A,B). In addition, we also identified that IL21‐AS1 and mIl21‐AS1 have no coding ability of protein or polypeptide (Figure S4C).

**FIGURE 3:**
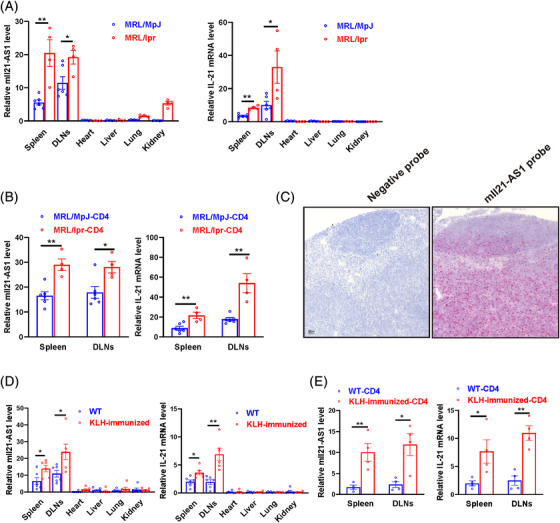
The expression of mIl21‐AS1 in lupus‐prone mouse model and keyhole limpet haemocyanin (KLH) immunized mice model. (A) RT‐qPCR was used to detect the expression of mIl21‐AS1 and IL21 mRNA in the heart, lung, liver, kidney, spleen and lymph nodes of MRL/lpr lupus model mice (16 weeks, *n* = 4) and MRL/MpJ (16 weeks, *n* = 6) mice. (B) RT‐PCR detected expression of mIl21‐AS1 and IL21 mRNA in CD4^+^ T cells of spleen and lymph nodes in MRL/lpr lupus model mice (*n* = 4) and MRL/MpJ mice (*n* = 6). (C) RNA‐scope detected the expression and distribution of mIl21‐AS1 in the lymph nodes of MRL/lpr lupus model mice. Scale bar: 50 μm. (D) RT‐qPCR was used to detect the expression of mIl21‐AS1 and IL21 mRNA in the heart, liver, lung, kidney, spleen and lymph nodes of KLH‐immunized mice (*n* = 6) and C57BL/6J control mice (*n* = 6). (E) RT‐qPCR detected expression of mIl21‐AS and IL21 mRNA in CD4^+^ T cells of spleen and lymph nodes in C57BL/6J control mice (*n* = 4) and KLH‐immunized mice (*n* = 4). Data were shown as the mean ± SEM. *p < .05, **p < .01, ***p < .001 relative to controls. *p*‐Value was determined using two‐tailed Student's *t*‐tests.

To explore the expression of mIl21‐AS1 and IL21 in different tissues of lupus‐like mouse model (MRL/Lpr), total RNA of heart, lung and so forth from MRL/Lpr mice and MRL/MpJ control mice were extracted. The results demonstrated that mIl21‐AS1 and IL21 were mainly expressed in the spleen and lymph nodes of mice, which were obviously higher in MRL/Lpr mice compared with MRL/MpJ mice (Figure 3A). Furthermore, we isolated CD4^+^ T cells from spleen of MRL/Lpr and MRL/MpJ mice and detected mIl21‐AS1 and IL21 expression. Similarly, the expression of mIl21‐AS1 and IL21 was significantly increased in splenic CD4^+^ T cells of MRL/Lpr compared with MRL/MpJ control mice (Figure 3B). Tissue in situ hybridization by RNA‐scope also confirmed the expression of mIl21‐AS1 in lymph nodes of MRL/Lpr (Figure 3C). Moreover, we also investigated the expression of mIl21‐AS1 and IL21 in different tissues of mice immunized with keyhole limpet haemocyanin (KLH). In comparison with wild‐type mice, the increased expressions of mIl21‐AS1 and IL21 were identified in spleen and lymph nodes and CD4^+^ T cells in KLH‐immunized mice (Figure 3D,E).

### 2.3 IL21‐AS1 regulates IL21 expression and Tfh cell differentiation

**FIGURE 4:**
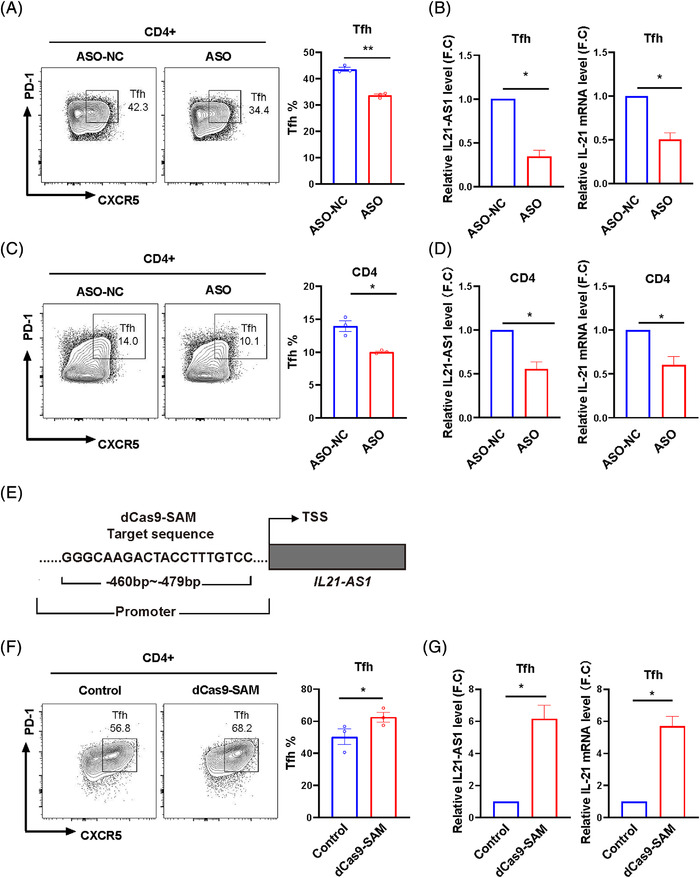
IL21‐AS1 regulates IL21 expression and Tfh cell differentiation. (A and B) Naïve CD4^+^ T cells from healthy donors were transfected with anti‐sense oligonucleotide (ASO), and negative controls (ASO‐NC) were differentiated into Tfh cells under Tfh‐polarization condition in vitro. Flow cytometry (FCM) detected the percentage of Tfh cells (CD4^+^CXCR5^+^PD1^+^) and RT‐qPCR detected the expression of IL21 and IL21‐AS1 in ASO group and ASO‐NC on day 3. (C and D) FCM detected the percentage of Tfh cells and RT‐qPCR detected the expression of IL21 and IL21‐AS1 in CD4^+^ T cell of systemic lupus erythematosus (SLE) patients transfected with ASO and ASO‐NC groups. (E) dCas9‐SAM targeting sequence at the promoter of IL21‐AS1. Naïve CD4^+^ T cells from healthy donors were transfected with IL21‐AS1 specific dCas9‐SAM, and negative controls (control) were differentiated into Tfh cells under Tfh‐polarization condition in vitro. (F and G) FCM detected the percentage of Tfh cells and RT‐qPCR detected the expression of IL21 and IL21‐AS1 in dCas9‐SAM group and control group. Data are representative of three independent experiments (mean ± SEM, *n* = 3). *p < .05, **p < .01, ***p < .001 relative to controls. *p*‐Value was determined using two‐tailed paired‐samples *t*‐test.

As IL21‐AS1 expression was increased significantly in Tfh cells and correlated with IL21 expression, we investigated whether IL21‐AS1 regulated IL21 expression and Tfh cells differentiation. Anti‐sense oligonucleotide (ASO) was applied to repress IL21‐AS1 expression. Naïve CD4^+^ T cells were purified and transfected with ASO targeting IL21‐AS1 (ASO) and negative control ASO (ASO‐NC). After transfection, cells were induced to Tfh cells under Tfh‐polarization culture condition for 3 days. IL21 expression and the percentage of Tfh cells were detected by RT‐qPCR and flow cytometry (FCM), respectively. Compared with the negative control, IL21‐AS1 and IL21 expressions were inhibited, and the proportion of Tfh cells was decreased in induced Tfh cells transfected with ASO (Figure 4A,B). Moreover, we also detected whether IL21‐AS1 contributed to the increased IL21 expression and Tfh cells proportion in SLE patients. We isolated total CD4^+^ T cells to transfect with ASO. We found that ASO could down‐regulate the mRNA levels of IL21‐AS1 and IL21 and reduce the proportion of Tfh cells in SLE CD4^+^ T cells (Figure 4C,D).

Recently, a few approaches for Cas9‐mediated transcriptional activation have been applied to increase gene transcript. To investigate whether the overexpression of IL21‐AS1 can promote IL21 expression and Tfh cells differentiation, we activated IL21‐AS1 expression by dCas9‐SAM transcriptional activation system (Figure 4E) in naïve CD4^+^ T cells in Tfh cells polarization condition in vitro. The results showed that dCas9‐SAM could activate IL21‐AS1 expression, thereby promoting IL21 expression and Tfh cells differentiation compared with negative control (Figure 4F,G). Together, these results indicate that IL21‐AS1 promotes IL21 expression and Tfh cells differentiation.

### 2.4 IL21‐AS1 binds to *IL21* promoter and hnRNPU protein in Tfh cells

**FIGURE 5:**
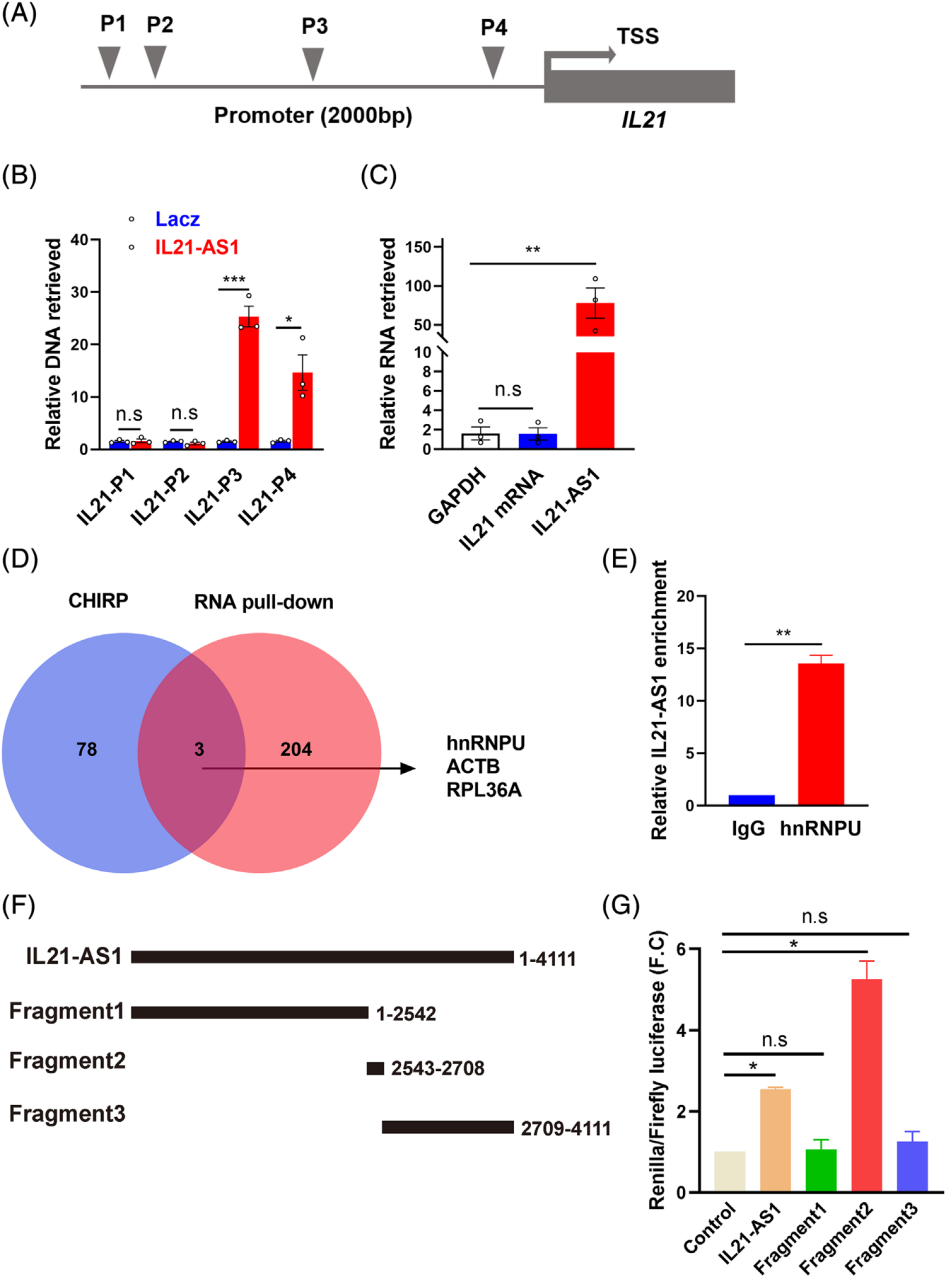
IL21‐AS1 binds to *IL21* promoter and heterogeneous nuclear ribonucleoprotein U (hnRNPU) protein in Tfh cells. (A) The position of the primers in the promoter region of *IL21* used in the chromatin isolation by RNA purification (ChIRP)‐qPCR. (B) The enrichment level of IL21‐AS1 was detected in the *IL21* gene promoter region by qPCR. (C) The enrichment of IL21‐AS1 at IL21 mRNA was detected by qPCR. (D) The shared binding proteins identified by ChIRP and RNA pull‐down. (E) RNA immunoprecipitation (RIP)‐qPCR analysed hnRNPU protein binding IL21‐AS1 by antibody of hnRNPU and isotype control IgG. (F) Length and location of truncated bodies used in dual‐luciferase reporter assay. (G) Analysis of Renilla/Firefly luciferase activities among groups. Data are representative of three independent experiments (mean ± SEM, *n* = 3). *p < .05, **p < .01, ***p < .001 relative to controls. *p*‐Value was determined using two‐tailed Student's *t*‐tests.

As IL21‐AS1 is positively correlated with mRNA level of IL21, we postulate that IL21‐AS1 regulates IL21 expression at transcription level. To uncover the underlying mechanism, we designed the biotin‐labelled ASO probe of IL21‐AS1 and performed the chromatin isolation by RNA purification (ChIRP) experiment in Tfh cells. The probes for ChIRP experiment were listed in Table S3. DNA and RNA were retrieved for qPCR analysis. Figure 5A shows the potential binding sequence of IL21‐AS1 and the locations of four primers for qPCR in the promoter region of *IL21* gene (Figure 5A). The results showed that IL21‐AS1 anti‐sense probe enriched significantly in the proximal promoter of *IL21* gene (P3 and P4) compared with negative Lacz probe but did not enrich in reference gene GAPDH DNA and IL21 mRNA (Figure 5B,C), indicating that IL21‐AS1 can bind to the promoter region of *IL21* gene to regulate *IL21* transcription.

Further, we identified the protein binding with IL21‐AS1 in Tfh cells. We first retrieved proteins that IL21‐AS1 anti‐sense probe enriched in ChIRP experiment and identified proteins by mass spectrometry (MS) (Figure S5A, Table S4). In addition, we also performed RNA pull‐down and MS experiments to identify proteins that pulled down by biotin–streptavidin‐labelled IL21‐AS1 oligonucleotides as probes (Figure S5B, Table S5). According to the results of two MS analyses, hnRNPU was identified in both ChIRP and RNA pull‐down experiment, indicating IL21‐AS1 binds with hnRNPU protein in Tfh cells (Figure 5D).

Previous study has shown that hnRNPU could enhance the function of Blnc1/EBF2 ribonucleoprotein complex and physically interact with lncRNA Blnc1 in human and mouse. To verify the binding of hnRNPU and IL21‐AS1, we performed the RNA immunoprecipitation (RIP) by hnRNPU antibody and five lncRNAs including UCA1, MIR100HG, lincRNA‐EPS, lincRNA‐AK023096 and ELF3‐AS1 were used as negative controls.^15^, ^16^, ^17^, ^18^, ^19^ IL21‐AS1 was identified in RNA enriched by hnRNPU antibody, but no significant enrichment of the five lncRNAs, which confirmed the interaction between IL21‐AS1 and hnRNPU protein in Tfh cells (Figures 5E and S6). We predicted a binding fragment (2543–2708 nt) with hnRNPU protein in IL21‐AS1 sequence by CatRAPID software (Figure S5C). To verify the function of binding fragment predicted by CatRAPID software, we co‐transfected dual‐luciferase reporter vector with *IL21* promoter fragment and pcDNA3.1 vector containing full‐length or truncated bodies of IL21‐AS1 into HEK 293T cells. The relative luciferase assay showed that IL21‐AS1 full‐length and fragment 2 (2543–2708 nt) could increase the ratios of Renilla/Firefly luciferase activities, suggesting IL21‐AS1, especially fragment 2 containing hnRNPU binding site, could promote the activation of *IL21* promoter significantly (Figure 5F,G).

### 2.5 IL21‐AS1 binding with hnRNPU and CBP proteins regulates the epigenetic modifications in *IL21* promoter

**FIGURE 6:**
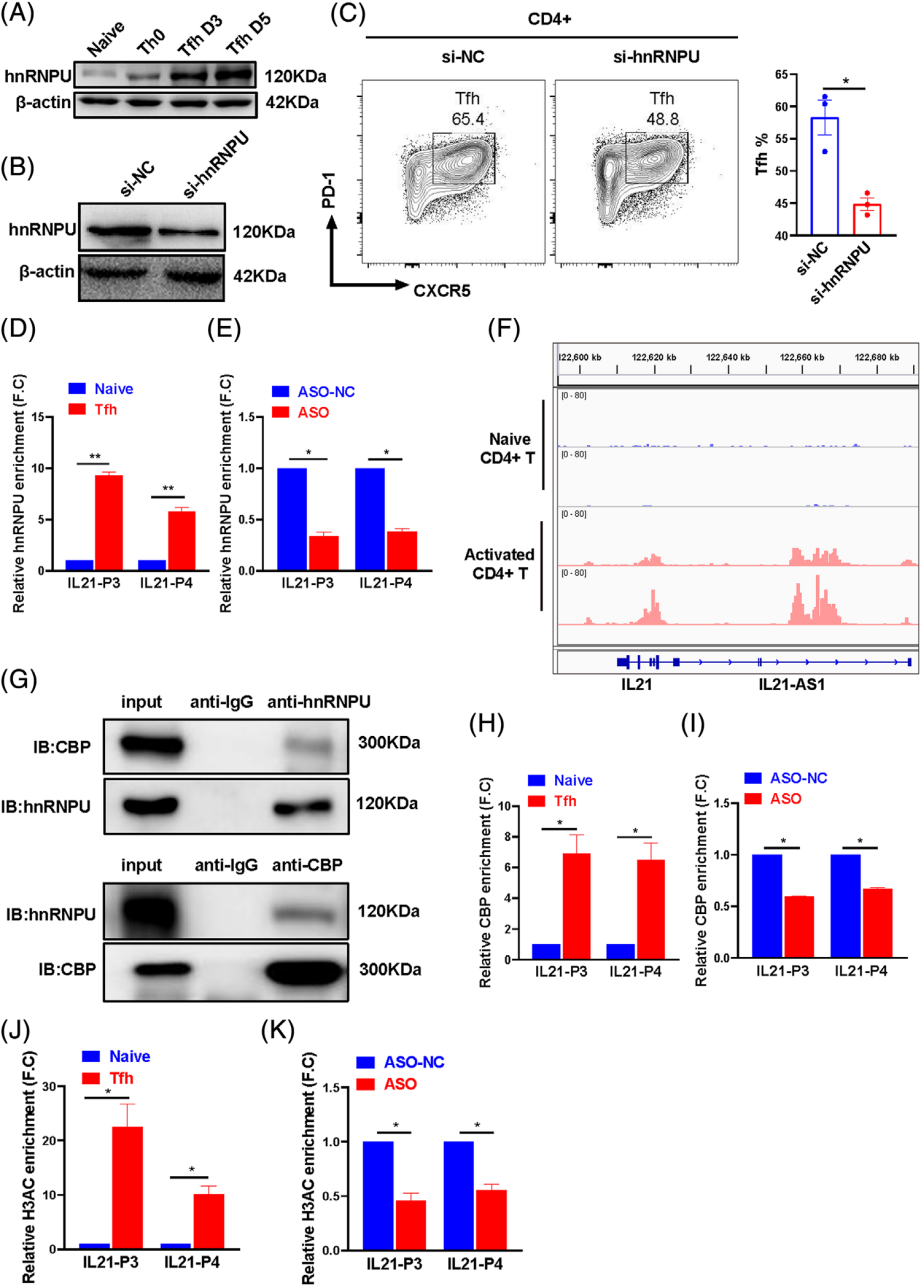
IL21‐AS1 binding with heterogeneous nuclear ribonucleoprotein U (hnRNPU) regulates the epigenetic modifications. (A) Western blot detected the expression of hnRNPU protein in induced Tfh cells. (B) Western blot was used to verify the interference effect of si‐hnRNPU. (C) Representative flow diagram of Tfh cells and statistical analysis of Tfh cells proportions in siRNA‐hnRNPU group and siRNA‐control group. (D) ChIP‐qPCR analysed the enrichment level of hnRNPU in the *IL21* gene proximal promoter region with hnRNPU antibody and IgG antibody in naïve CD4^+^ T and Tfh cell. (E) ChIP‐qPCR analysed the enrichment level of hnRNPU in the *IL21* gene proximal promoter region in IL21‐AS1 anti‐sense oligonucleotide (ASO) group and control group. (F) H3K27 acetylation peak in the promoter region of *IL21* according to public data (GSE177090, GSE177374, GSE177639 and GSE177926). (G) Co‐IP and western blot detected the interaction between hnRNPU and CREB‐binding protein (CBP) in Tfh cells. (H) ChIP‐qPCR analysed the enrichment level of CBP in the *IL21* gene proximal promoter region in naïve CD4^+^ T and Tfh cell. (I) ChIP‐qPCR analysed the enrichment level of CBP in the *IL21* gene proximal promoter region in IL21‐AS1 ASO group and control group. (J) ChIP‐qPCR detected the H3 acetylation (H3ac) level in *IL21* proximal promoter region in naïve CD4^+^ T cells and Tfh cells. Data are representative of three independent experiments (mean ± SEM, *n* = 3). (K) ChIP‐qPCR detected the H3ac level in *IL21* proximal promoter region in IL21‐AS1 ASO group and control group. *p < .05, **p < .01, ***p < .001 relative to controls. *p*‐Value of Tfh cells between control group and IL21‐AS1 ASO group was determined using two‐tailed paired‐samples *t*‐test, and others were determined using two‐tailed Student's *t*‐tests.

The role of hnRNPU protein in Tfh cells differentiation remains unclear. We first detected the protein level of hnRNPU in naïve CD4^+^ T cells, Th0 and the induced Tfh cell. The results demonstrated that hnRNPU protein levels were increased significantly in the induced Tfh cells on day 3 and 5, but not in Th1/2/17/Treg cells, compared with Th0 and naïve CD4^+^ T cells (Figures 6A and S7A). In addition, we inhibited the expression of hnRNPU and induced Tfh cells differentiation. The results showed that an interference of hnRNPU reduced the differentiation of Tfh cells, indicating that hnRNPU is involved in regulating Tfh cells differentiation (Figure 6B,C).

hnRNPU protein has the affinity for both RNA and DNA, which is involved in maintaining 3D genome architecture.^20^ To demonstrate the mechanism of IL21‐AS1 regulating *IL21* transcription, we detected the binding of hnRNPU in *IL21* promoter. Compared with naïve CD4^+^ T cells, we found a significantly increased enrichment of hnRNPU protein in the proximal promoter of *IL21* in Tfh cells (Figure 6D). Moreover, in comparison with negative control, the enrichment of hnRNPU protein in *IL21* promoter was reduced significantly in CD4^+^ T cells with IL21‐AS1 knock‐down (Figure 6E), suggesting the binding of hnRNPU protein in *IL21* promoter was dependent on IL21‐AS1 in CD4^+^ T cells.

According to the data of ROADMAP, there are some active histone markers such as histone H3K27 acetylation in the promoter region of *IL21* gene in activated CD4^+^ T cells (Figure 6F). Previous study showed that hnRNPU interacts with p300 to regulate transactivation of EGR1.^21^ However, our results showed that the enrichment of CBP in *IL21* promoter, but not the enrichment of p300, was decreased significantly in Tfh cells with IL21‐AS1 knock‐down compared with negative control. Moreover, the enrichment of CBP at the *IL21* promoter was increased in Tfh cells compared with naïve CD4^+^ T cells (Figures 6H,I and S7B). The histone acetylation of *IL21* promoter was increased significantly in Tfh cells compared with naïve CD4^+^ T cells, and the H3ac levels in *IL21* promoter were decreased significantly in Tfh cells with IL21‐AS1 knock‐down compared with negative control, which suggested that IL21‐AS1 regulates histone acetylation level of *IL21* promoter (Figure 6J,K).

### 2.6 mIl21‐AS1 promotes Tfh cell differentiation and humoral immune response in mice

**FIGURE 7:**
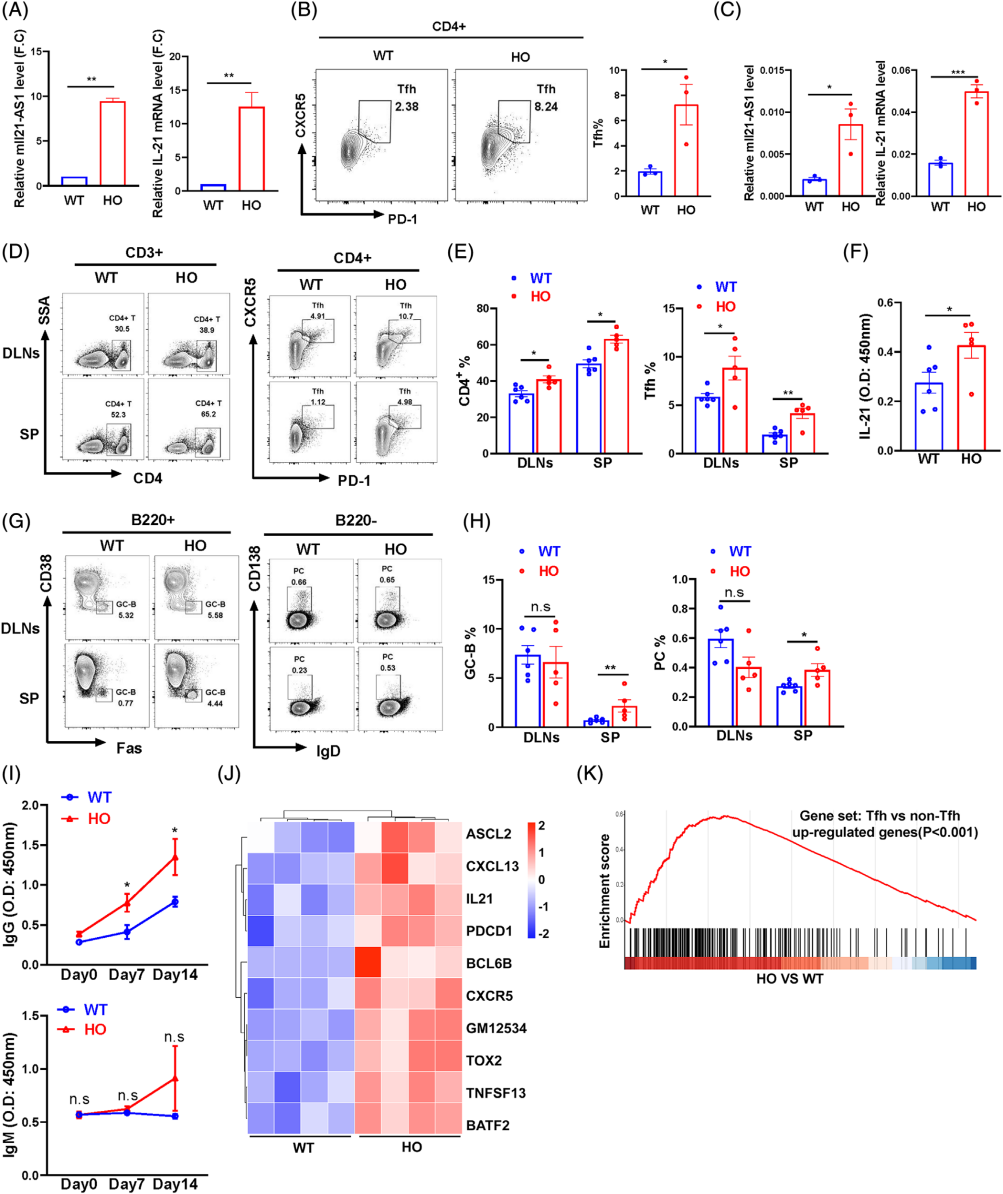
mIl21‐AS1 promotes Tfh cell differentiation and humoral immune response in mice. (A) RT‐qPCR detected the expression of IL21 and mIl21‐AS in CD4^+^ T cells of spleen and lymph nodes from wild‐type mice (WT, *n* = 3) and homozygous mice (HO, *n* = 3). (B and C) Splenic CD4^+^ T cells from HO and WT mice were stimulated with anti‐CD3 and anti‐CD28 antibodies for 2 days (*n* = 3), flow cytometry (FCM) detected the percentage of Tfh (CD4^+^CXCR5^+^PD1^+^) cells and RT‐qPCR detected mIl21‐AS and IL21 level. (D) Representative flow diagram of CD4^+^ T, Tfh (CD4^+^CXCR5^+^PD1^+^) in draining lymph nodes (dLNs) and spleens of wild‐type (*n* = 6) and homozygous mice (*n* = 5) immunized by keyhole limpet haemocyanin (KLH). (E) Statistical analysis of the percentages of CD4^+^ T, Tfh cells in wild‐type (*n* = 6) and homozygous mice (*n* = 5). (F) IL21 levels in serum of HO and WT mice immunized by KLH on day 14 were detected by ELISA. (G) Representative flow diagram of germinal centre (GC)‐B (B220^+^Fas^+^CD38^−^), plasma (B220^−^IgD^−^CD138^+^) in dLNs and spleens from wild‐type (*n* = 6) and homozygous mice (*n* = 5) immunized by KLH. (H) Statistical analysis the percentages of GC‐B, plasma cells from wild‐type (*n* = 6) and homozygous mice (*n* = 5). (I) Total anti‐IgG, anti‐IgM levels in serum of HO and WT mice immunized by KLH on day 0, 7 and 14. (J) RNA‐seq analysis of Tfh‐related gene expression in isolated CD4^+^ T cells from KLH‐immunized wild‐type (*n* = 4) and homozygous mice (*n* = 4). (K) The up‐regulated genes in CD4^+^ T cells from HO mice were compared with the up‐regulated genes signature in Tfh cells from published data (GSE16697). Data were shown as the mean ± SEM. *p < .05, **p < .01, ***p < .001 relative to controls. *p*‐Value was determined using two‐tailed Student's *t*‐tests.

To explore the role of mIl21‐AS1 in IL21 expression and Tfh cells differentiation, we constructed knock‐in mice with mIl21‐AS1 insertion at Hipp11 locus by CRISPER‐cas9 system (Figure S8A,B). We isolated CD4^+^ T cells of homozygous mIl21‐AS1^+/+^ mice (HO), heterozygous mIl21‐AS1^+/−^ mice (HE) and wild‐type mice (WT) and compared the expression level of mIl21‐AS1. In comparison with WT mice, mIl21‐AS1 expression was higher in HO and HE mice, indicating a successful mice model with mIl21‐AS1 overexpression (Figures 7A and S8C). Furthermore, CD4^+^ T cells from spleen of HO and WT mice were activated in vitro. The results showed that IL21 expression level and the proportion of Tfh cells were higher in activated CD4^+^ T cells from HO mice than WT mice (Figure 7B,C).

Moreover, we stimulated HO mice and WT mice with KLH by intraperitoneal injection to observe the effect of mIl21‐AS1 on the differentiation of Tfh cells and humoral immune response in vivo. On day 14, we isolated single cells from draining lymph nodes (dLNs) and spleen of mice for FCM analysis. The results showed that the percentages of both total CD4^+^ T cells and Tfh cells were increased significantly in dLNs and spleen of HO mice compared with WT mice (Figure 7D,E), and the level of IL21 protein in serum was higher in HO mice than that in WT mice (Figure 7F). The proportion of naïve CD4^+^ T cells was decreased, and the proportion of memory CD4^+^ T cells was increased in the spleen of HO mice compared to WT mice, but no significant difference in other Th cells subsets, including Th1, Th2 and Th17 cells (Figure S8D,E). In addition, we also found that the proportion of IFN‐γ^+^CD4^−^ T cells was increased, and the percentage of CD8^+^ T cells was decreased significantly in mice immunized with KLH (Figure S8F–I). The proportions of germinal centre (GC)‐B cells and plasma cells were also increased significantly in the spleen of HO mice compared with WT mice (Figure 7G,H). Moreover, we measured the levels of IgG and IgM in the serum of HO mice and WT mice. The results showed that the IgG levels on day 7 and 14 during KLH immunization experiment were increased significantly in HO mice compared with WT mice, but no significant difference in IgM level (Figure 7I). Moreover, we isolated CD4^+^ T cells from spleen and dLNs of HO and WT mice immunized by KLH on day 14 and detected the mRNA expression profiles by RNA‐seq. We identified 1576 up‐regulated genes and 526 down‐regulated genes (|log_2_FC| > .667, *p*‐value <.05, Figure S9A and Table S6), in which besides mIl21‐AS1 (Gm12534), the expression levels of some Tfh related genes such as IL21, CXCR5, PDCD1, Ascl2^22^ and TNFSF13^23^ were up‐regulated significantly in CD4^+^ T cells of dLNs and spleen in HO mice compared with WT mice (Figure 7J). Furthermore, we compared the up‐regulated gene expression profile in HO mice with the published data (GSE16697), which included the increased genes in Tfh cells compared with non‐Tfh cells. Gene set enrichment analysis showed that the up‐regulated genes in CD4^+^ T cells of HO mice were highly consistent with the expression of Tfh cell signature genes (Figure 7K). Together, these results indicate that mIl21‐AS1 has similar functions as IL21‐AS1 in promoting Tfh cells differentiation and humoral immune response. Gene ontology enrichment analysis showed that up‐regulated genes in HO mice were enriched significantly in wounding response, oxidative stress response, positive regulation of cell activation, leukocyte migration and so forth (Figure S9B), which suggested that IL21‐AS1 may be involved in regulating other biological processes in vivo.

In addition, we also stimulated HO mice and WT mice with NP‐KLH. The same changes of naïve CD4^+^ T cells, memory CD4^+^ T cells, Tfh cells and GC‐B cells were observed in HO mice compared with WT mice (Figure S10A–D). Furthermore, we also found that more NP_2_‐ and NP_27_‐specific IgG1 antibodies at day 14 and IgM antibodies at day 28 were produced in HO mice than those in WT mice (Figure S10E,F).

### 2.7 mIl21‐AS1 overexpression aggravates the lupus‐like phenotypes of mice

**FIGURE 8:**
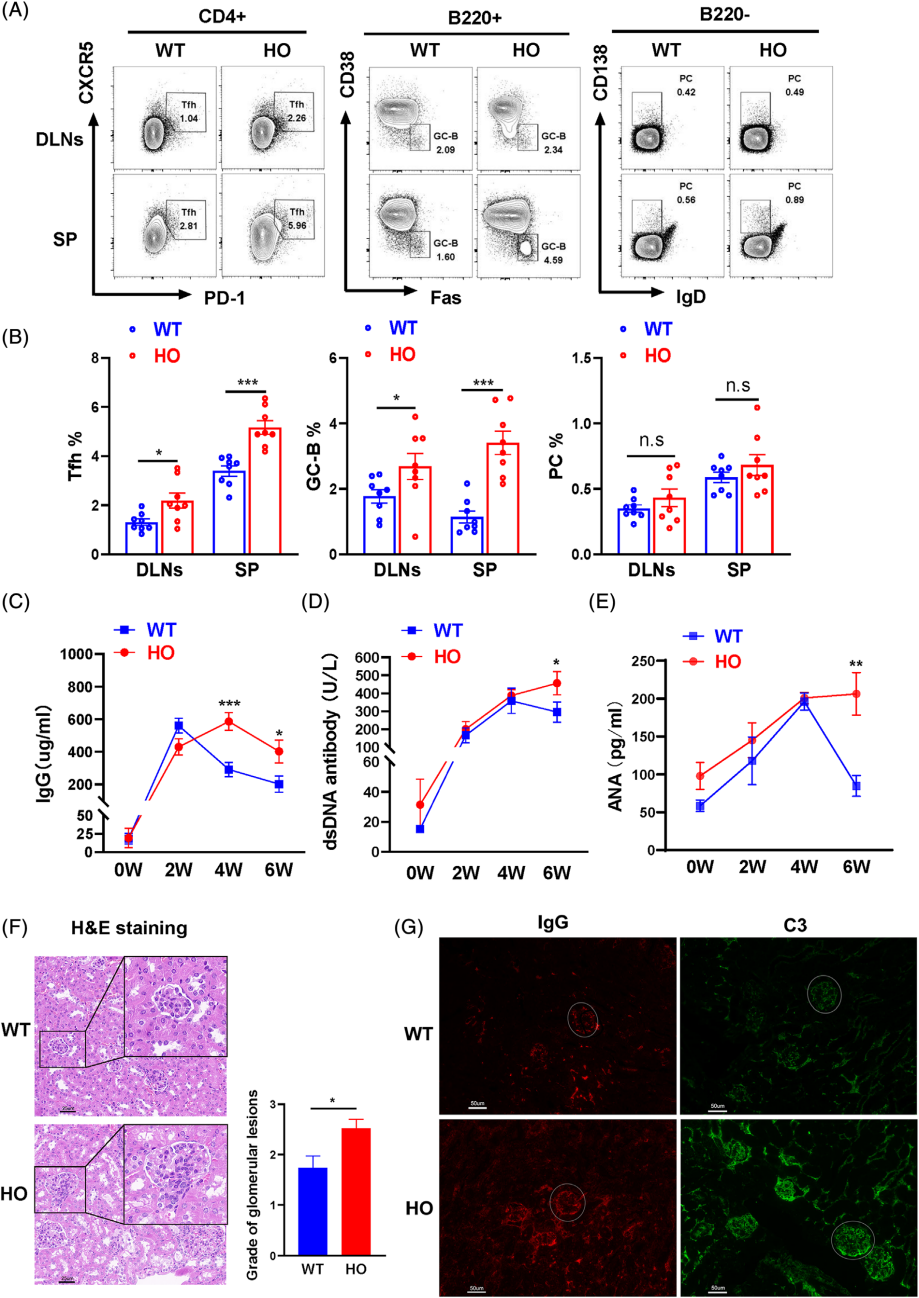
mIl21‐AS1 overexpression aggravates the lupus‐like phenotypes in chronic graft versus host disease (cGVHD) model. (A) Representative flow diagram of Tfh cells, germinal centre (GC)‐B cells, and plasma cells (PC) in HO and WT cells‐induced cGVHD mice (*n* = 8 each group). (B) Statistical analysis of the percentages of Tfh, GC‐B and PC cells. (C–E) The serum levels of anti‐total IgG antibody, anti‐dsDNA antibody and antinuclear antibody (ANA) in HO and WT cells‐induced cGVHD models were determined by ELISA on weeks 0 and 6 (*n* = 8 each group). (F) H&E staining and the grades of glomerular lesions in the kidney of cGVHD models induced by HO and WT mice (*n* = 8 each group). Scale bar: 25 μm. (G) C3 and IgG deposition in kidney of cGVHD models induced by HO and WT mice. Scale bar: 50 μm. Data are shown as the mean ± SEM. *p < .05, **p < .01, ***p < .001 relative to controls. *p*‐Value was determined using two‐tailed Student's *t*‐tests.

Chronic graft versus host disease (cGVHD) is a recognized lupus model that has been used in previous publication to study SLE.^24^ To explore the role of mIl21‐AS1 in lupus, a total of 5 × 10^7^ CD8^+^ cells‐depleted lymphocytes from HO and WT mice were injected into B6D2F1 mice via the tail vein respectively. At 6 weeks, we detected the autoimmune responses and renal damage in mice. The results showed that the percentages of Tfh cells in dLNs and spleen were significantly increased in HO mice compared with WT mice, and the proportion of GC‐B cells was also increased in the spleen of HO mice. The percentage of PC cells has an upward tendency but no statistical difference between HO and WT mice (Figure 8A,B). In addition, the naïve CD4^+^ T cells proportion was decreased, and the memory CD4^+^ T cells proportion was increased in spleen of HO mice. The Th2 proportion was elevated, and Th17 proportion has an upward tendency but no statistical difference in DLNs of HO mice (Figure S11A,B). Moreover, we found that the levels of IgG, dsDNA‐antibody and antinuclear antibody in serum were increased significantly in HO mice compared with WT mice (Figure 8C–E). Furthermore, we observed more severe renal damage with higher renal scores according to the H&E staining and more depositions of IgG and C3 in the kidney glomerulus of HO mice compared with WT mice (Figure 8F,G).

## 3 DISCUSSION

**FIGURE 9:**
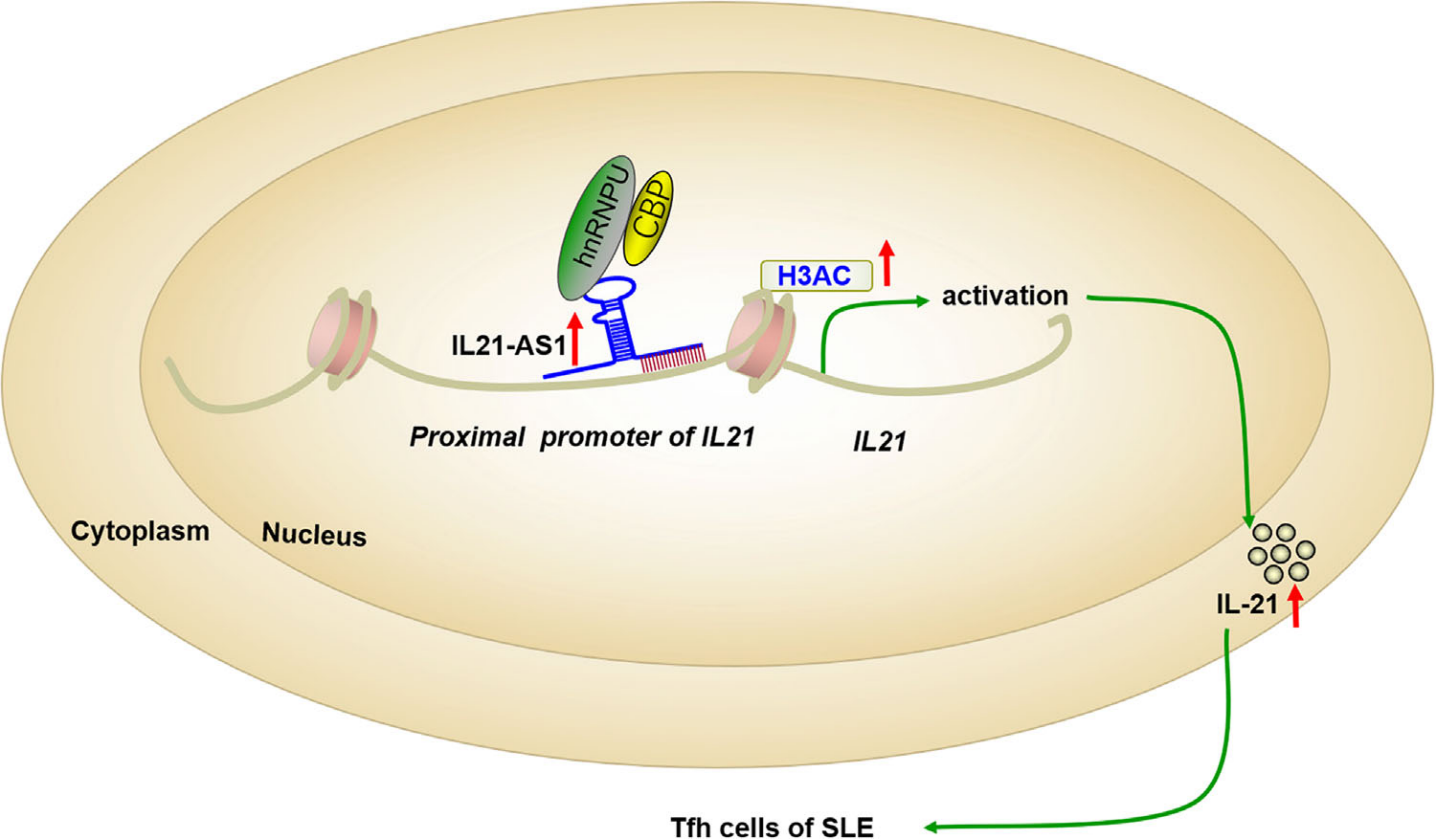
Model of IL21‐AS1 regulating IL21 expression and Tfh differentiation in systemic lupus erythematosus (SLE) patients. IL21‐AS1 was highly expressed in CD4^+^ T cells of SLE patients and Tfh cells, which directly binds to promoter of *IL21* gene and interacts with heterogeneous nuclear ribonucleoprotein U (hnRNPU) and CREB‐binding protein (CBP) protein to regulate H3 acetylation level in the promoter region of *IL21*. IL21‐AS1 overexpression promotes *IL21* transcription activation and the aberrant differentiation of Tfh cells in SLE patients, thereby exacerbating autoimmune phenotypes of SLE. Red arrow indicates up‐regulation of expression. Green line with arrow indicates positive regulation.

The expansion of circulating Tfh cells in SLE patients has been shown by previous studies, which were correlated with overproductions of autoantibodies and the SLEDAI scores of SLE.^25^, ^26^ However, the mechanism leading to the abnormal differentiation of Tfh cells is not clear yet. In this study, we identified and cloned a lncRNA IL21‐AS1 as a positive regulator of Tfh cells differentiation. IL21‐AS1 expression was significantly increased in SLE CD4^+^ T cells and induced Tfh cells. The results of this study demonstrated that IL21‐AS1 could promote Tfh cells differentiation via up‐regulation of target gene IL21 expression. In Tfh cells, IL21‐AS1 could bind to the promoter region of *IL21* gene, as well as recruite the protein complex of hnRNPU and CBP, which activate the transcription of *IL21* gene via increasing the histone H3ac. Importantly, we also cloned a mouse homologous lncRNA mIl21‐AS1, which also transcribed from the anti‐sense sequence of mouse Il21 gene and highly expressed in mouse Tfh cells. Overexpression of exogenous mIl21‐AS1 in vivo could promote the Tfh‐mediated humoral immune response and aggravate the lupus‐like disease phenotypes in cGVHD lupus model. Together, we identified a lncRNA regulating differentiation of Tfh cells, which is crucial in the pathogenesis of SLE (Figure 9).

Sequence conservation of lncRNA is rather poor, which is unlike with protein‐coding genes. Therefore, it is difficult to identify their homologous genes.^27^, ^28^ A lot of evidences indicated that lncRNAs tend to preserve their regulatory machinery rather than their transcribed sequence.^29^ Previous study reported that lncRNA NEAT1 is abnormally highly expressed in PBMCs of SLE patients and myeloid‐derived suppressor cells from MRL/lpr mice.^30^, ^31^ Here, we identified a mouse homologous lncRNA mIl21‐AS1 in mouse Tfh cells. mIl21‐AS1 is transcribed from the opposite direction to a sense protein‐coding mouse gene *Il21* and located in the 5′‐end of *Il21*, which suggests a similar function and regulatory machinery with human IL21‐AS1. Our results showed that mIl21‐AS1 can promote Tfh cells differentiation and humoral immune response as the in vitro and in vivo results indicated. Therefore, the identification of homologous mIl21‐AS1 indicates a high conservation and important role in biological process among different species as well as provides a convenience for investigating the function of lncRNA in vivo.

Although the critical roles of lncRNAs in the pathogenesis of some diseases, especially in cancer, have been reported, the lncRNAs are still rarely identified and studied in autoimmune diseases. Recent studies have reported the functions and mechanisms of some lncRNAs in SLE.^32^, ^33^ LncRNA expression profiling revealed a lot of differentially expressed lncRNAs in PBMCs, neutrophils and monocyte‐derived DC between SLE and normal controls, some of which were correlated with the severity of disease and type I interferon pathway in SLE.^34^, ^35^ Several lncRNAs are related to the activation of T cells in SLE. LncRNA GAS5 was reduced in SLE patients, including CD4^+^ T cells and plasma. Overexpression of GAS5 inhibited CD4^+^ T cells activation and reduced the self‐reactivity of SLE CD4^+^ T cells.^36^ A previous study reported that IL21‐AS1 expression was reduced in PMBCs of SLE and negatively correlated with SLEDAI score. In addition, they also found a decreased expression of IL21‐AS1 in CD4^+^ T cells of eight SLE patients compared to eight HCs.^37^ However, in this study, we observed an obvious increase of lncRNA IL21‐AS1 expression according to RNA‐seq data of CD4^+^ T cells in 12 SLE patients and 8 HCs. Furthermore, we validated the increased expression of lncRNA IL21‐AS1 in CD4^+^ T cells of 40 SLE patients compared with 40 HCs, which was positively correlated with IL21 gene expression in SLE patients and the SLEDAI scores. We speculated the discrepancy between the two studies may be due to complex cellular composition of PBMCs, different genetic backgrounds of patients, the very limited sample size of SLE patients in the previous study.

Recently, some studies reported the role of lncRNAs in the apoptosis, activation, proliferation and differentiation of effect T helper cells.^38^, ^39^, ^40^, ^41^ However, the role of lncRNAs in Tfh cells still need to be investigated. In this study, we found that IL21‐AS1 regulates IL21 expression. Both Tfh and Th17 could produce IL21, and IL21 has been proven to participate in promoting proliferation and differentiation of Tfh and Th17 cells, balancing helper T cell subsets, generation of B cells and differentiation into plasma cells, and boosting the immunoglobulin production.^10^ The elevated IL21 level is positively associated with Tfh cells, plasma cells, autoantibodies and disease activity in SLE patients.^42^ In this study, we found that overexpressing IL21‐AS1 promoted Tfh cells differentiation in mice immunized with KLH, meanwhile, the proportion of Th17 cells was increased mildly, and the proportions of Th1, Th2 and Treg cells have no significant change in mice immunized with KLH compared with wild‐type mice. Interestingly, results of our study showed that the percentage of IFN‐γ^+^CD4^−^ T cells was obviously elevated in mice immunized with KLH. As we uncovered that the proportion of CD8^+^ T cells was reduced, we inferred that IL21‐AS1 overexpression promotes the development of NKT cells. IL21 has been reported to strengthen NKT cell proliferation and enhances the cytotoxicity and IFN‐γ production by murine NK cells.^43^ In conclusion, the results of our study indicated the important role of IL21‐AS1 in adaptive immune, as well as innate immune.

cGVHD was induced in bm12 host mice as previously described.^44^ Mice were followed for serum autoantibody levels, which is a widely used model in study of SLE.^45^, ^46^, ^47^ Previous studies have reported that some processes were required for multiorgan system cGVHD, such as GC formation and immunoglobulin accumulation, in which Tfh cell and B cells play an essential and key role. Blocking mAbs for IL21/IL21R, ICOS/ICOS ligand, and CD40L/CD40 hindered GC formation and cGVHD.^48^, ^49^, ^50^ These findings indicated that cGVHD model is Tfh‐dependent. Therefore, we performed the cGVHD model through injecting the lymphocytes from HO and WT mice into B6D2F1 mice. Our results indicated that an overexpression of mIl21‐AS1 promoted the development of cGVHD model, which suggests that mIl21‐AS1 plays a crucial role in the development of SLE.

LncRNA regulates gene expression via multiple mechanisms, among which modulating epigenetic modifications and mRNA splice by some RNA binding proteins are important in the process.^51^ In our study, we identified that lncRNA IL21‐AS1 binds hnRNPU by RNA pull‐down and RIP assays. HnRNPU belongs to hnRNPs family, which concludes approximately 20 proteins and is separated into hnRNPAl‐U relying on the molecular weight.^52^ Interactions of lncRNAs and hnRNPs in regulating gene expression have been reported by that previous study, including transcriptional, post‐transcriptional levels or genomic structure changes.^20^, ^53^ HnRNPU also binds with Xist RNA, which was required for the accumulation of Xist RNA on the Xi.^54^ LBCS could inhibit SRY‐box 2 (SOX2) transcription via histone H3 lysine 27 tri‐methylation, in which LBCS acts as a scaffold through direct links with hnRNPK and EZH2.^55^ CBP and p300 are two paralogous lysine acetyltransferases (KATs), mediating histone acetylation. A previous report has indicated that HPSE eRNA bond to hnRNPU to facilitate its interaction with p300, which mediated the transactivation of EGR1.^21^ To verify whether IL21‐AS1 interacts with hnRNPU and p300 or CBP to regulate IL21 expression in Tfh cells, we repressed IL21‐AS1 expression using ASO in naïve CD4^+^ T cells under Tfh‐polarization condition and then detected the enrichments of p300 and CBP in *IL21* promoter. The results demonstrated that the enrichment of CBP in *IL21* promoter, but not the enrichment of p300, was decreased significantly in Tfh cells with IL21‐AS1 knock‐down compared with negative control, which suggested an interaction of IL21‐AS1 with CBP to regulate IL21 expression in Tfh cells. Therefore, those results demonstrate that IL21‐AS1 plays a vital role in regulating the aberrant modifications in Tfh cells as well as demonstrate the mechanism of IL21‐AS1 promoting *IL21* transcription and Tfh cells differentiation.

Taken together, our research identifies a lncRNA IL21‐AS1 and its mouse homologous lncRNA mIl21‐AS1 that promotes Tfh cells polarization and function through regulating IL21 expression. We provide new insights into the epigenetic mechanisms in Tfh cell differentiation and humoral immune and suggest potential clues for an intervention of systemic inflammation and autoimmune diseases.

## 4 MATERIALS AND METHODS

*Study subjects*: The study currently enrolls 42 patients (4 males and 38 females) who were diagnosed with SLE and 42 sex‐ and age‐matched healthy donors (5 males and 37 females), respectively, from the outpatient clinics and the medical staff at the Second Xiangya Hospital (Table S7). All patients met the ACR revised criteria.^56^ An assessment of disease activity was conducted by using the SLEDAI score.^57^ Human tonsil tissues were collected from the Second Xiangya Hospital. All participants have written informed consent.

*Mice*: Slack Company provided the MRL/MpJ mice, MRL/lpr lupus model mice and C57BL/6J wild‐type mice. The mIl21‐AS1 knock‐in mouse model was constructed by Saiye Guangzhou Biotechnology Co., Ltd. B2D6F1 mice were purchased from SPF (Beijing) Biotechnology Co., Ltd.

*In vitro differentiation of human T cells*: The anti‐CD3 antibody was pre‐coated in cold overnight, and the prepared cells were used to induce T subsets under different polarization conditions. The specific conditions were listed in Table S8.

*Transfection of the IL21‐AS1 Anti‐sense oligonucleotide (ASO)*: Electroporation kits for human T cells (Lonza) were used to perform transfection of sorted cells with 200 nM ASO. The target sequence of ASO is CCTCACGGAAGGCCAAAGAC, which locates at the exon2 of IL21‐AS1.

*Overexpression of IL21‐AS1 by dCas9‐SAM gene activation system*: IL21‐AS1 activation in purified naïve CD4^+^ T cells was performed with the dCas9‐SAM gene activation method. This system contains three lentivirus plasmids, dCas9‐VP64, MS2‐P65‐HSF1 and small guide RNA (sgRNA). Those three lentiviruses were applied to naïve CD4^+^ T cells in Tfh‐induced condition. dCas9‐VP64, MS2‐P65‐HSF1 and empty vector without sgRNA as control. Cells were harvested 3 days after infection for further experiments.

*RNA isolation and RT‐qPCR*: TRIzol (Invitrogen) was applied to extract RNA. Then, cDNA was generated by using a kit (Takara). The expression of target gene amplification signal was analysed with Roche system. The used primer sequences were provided in Table S9.

*Western blotting and coimmunoprecipitation (Co‐IP)*: Cells were lysed with RIPA buffer, and BCA kit (Thermo Scientific) was used to determine the concentration of protein. The antibodies used as follows: anti‐FLAG (1:1000, 14793, Cell Signaling Technology), anti‐CBP (1:1000, ab253202, abcam), anti‐hnRNPU (1:2000, MA1‐24632, Thermo Scientific) and anti β‐actin (1:1000, 4970, Cell Signaling Technology). For Co‐IP, anti‐hnRNPU antibody (1:100) and anti‐CBP antibody (1:100) were used to immunoprecipitate hnRNPU and CBP, respectively, before western blotting. The immunoprecipitated proteins were further isolated by the following instructions of Co‐IP assay kit (Thermo Fisher Scientific) and then detected by western blotting.

*Chromatin immunoprecipitation (ChIP)‐qPCR*.: Samples were collected and cross linked with 1% formaldehyde, and then 1% glycine to stop. The collected samples were lysed and sonicated. A volume of 5 μl anti‐acetyl histone H3 (Active Motif) or 5 μl anti‐CBP (abcam), 5 μl anti‐hnRNPU (Thermo Scientific) or normal IgG (Millipore) were added into the sheared DNA overnight. Normal IgG was used as a negative control. After decrosslinking, the purified DNA was measured by qPCR. The primers are shown in Table S9.

*KLH immunization*: Age‐matched HO and WT mice were treated with KLH (.5 mg/ml) (Biosearch Technologies) emulsified in Complete Freund's adjuvant (100 μl per mouse) by means of subcutaneous injection, subcutaneous administration at the base of the tail. After immunization for 1 week, the second injection with the equal amount of KLH was performed to enhance immune response 7 days later.

*NP‐KLH immunization*: Age‐matched HO and WT mice were immunized with NP‐KLH (2 mg/ml) in Alum (100 μl per mouse), subcutaneous administration at the base of the tail. The second injection with the equal amount of NP‐KLH was performed to enhance immune response 7 days later. All mice were sacrificed at day 28.

### 4.1 cGVHD lupus model

A total of 5 × 10^7^ CD8^+^ T cells‐depleted lymphocytes of HO and WT cells were injected into B2D6F1 mice via tail veins, urine and serum were collected every 2 weeks, by the end of 6 weeks, mice were sacrificed for further experiment, urine protein was detected by colorimetric assay strips (URIT), anti‐total IgG, anti‐ANA antibodies and anti‐dsDNA antibodies were detected by ELISA kits (CUSABIO, China), and kidney slides were used to visualized total IgG and C3 deposition.

### 4.2 5′ and 3′ rapid amplifications of cDNA ends (RACE)

The 5′ and 3′ RACEs of IL21‐AS1 were performed by following instructions of the SMARTer RACE5′/3′kit (Clontech). Total RNA extracted from human Tfh cell and mouse Tfh cell in vitro culture was used, and 1 μg total RNA from each sample were used for RACE. The specific primers for 5′ and 3′ RACE were designed based on known sequence information, shown in Table S10.

*Northern blot*: Northern blot was performed by using DIG labelling kit (Roche). In brief, the total RNA was electrophoresed and fixed to the NC membrane, for fixing, ultraviolet light was used to cross link the RNA, and then the membrane was incubated in ULTRAhyb‐oligo Hy‐ bridization buffer and hybridized with the IL21‐AS1‐specific oligonucleotide probes labelled with digoxigenin‐ddUTP. The probe was designed by Thermo Fisher Scientific, shown in Table S11.

*RNA‐FISH*: We used the RNA‐FISH to study the subcellular distribution of IL21‐AS1. Fluoresce‐conjugated IL21‐AS1 probes labelled with Cy3 and FISH kits were generated from RiboBio (China). Briefly, 4% paraformaldehyde (supplementing .5% TritonX‐100) was used to fix our samples (30 min). The fixed cells were incubated with IL21‐AS1 probes in hybridization buffer at 37°C overnight. Nuclei were stained with DAPI. Nuclei were counter‐stained with DAPI. We used a confocal microscope to take images.

*RNA immunoprecipitation (RIP)*: The whole procedure followed RIP kit (Millipore). Briefly, The Tfh cells of 1 × 10^7^ were lysed and sonicated on ice after sonication, the supernatant was aliquoted and incubated with 5 μl anti‐hnRNPU antibody overnight at 4°C. Reverse transcription was performed to generate cDNA, and IL21‐AS1 level was determined by RT‐qPCR.

*RNA pull‐down*: RNA pull‐down assay was carried out following an instruction of the RNA‐Protein Pull‐Down Kit (Millipore). Briefly, biotinylated IL21‐AS1 or negative control RNAs was incubated with whole cell extracts separated from 1 × 10^7^ Tfh cells at 4°C overnight. The complexes of the biotinylated IL21‐AS1 and proteins were purified using streptavidin‐agarose at 4°C overnight. Finally, MS analysis was used to detect the RNA‐protein binding mixtures.

*RNA‐scope in situ hybridization*: In this study, we detected the mIl21‐AS1 expression in heart, liver, lung, kidney and lymph node by RNA‐scope 2.0 following Advanced Cell Diagnostics kit. Each step was performed following standard procedures (ACD). In brief, the tissue volume was the soybean grain‐sized and fixed in neutral formalin (10%) for 24 h and then sliced tissue sections (4 μm thick) and placed on a glass slide. Slides were deparaffinized in xylene for 1 h at 70°C for deparaffinized and followed by dehydration in an ethanol series. A citrate buffer at a boiling temperature incubated for 15 min, and deionized water rinsed tissue sections three times and protease digestive tissue section at 40°C for 30 min. Tissue section was then incubated in order at 40°C with five kinds of hybridization buffers following the standard procedure of the kit. The specimens were counter‐stained with haematoxylin. The slides were observed under a microscope (Olympus).

### 4.3 Flow cytometry (FCM)

For surface marker, cells were incubated in cold for 30 min, and for intracellular markers, cells were stained with antibodies in cold for 45 min after fixation and permeabilization. For human sample, anti‐CD4 (BioLegend, 317408), anti‐PD1 (BioLegend, 379208), anti‐CXCR5 (BD, 562781); for mouse sample, anti‐CD4 (BioLegend, 100408), anti‐CD279 (BioLegend, 135210), biotin anti‐CXCR5 (BD, 551960) and PE conjugated streptomycin (BD, 554061), anti‐CD3 (BioLegend, 100312), anti‐CD8 (BD, 551162), Zombie NIR (BD, 423106), anti‐IFN‐γ (BD, 554412), anti‐IL‐4 (BioLegend, 404118), anti‐IL‐17A (BD, 560666), anti‐mouse B220 (BioLegend, 103222), anti‐IgD (BD, 553510), anti‐CD138 (BioLegend, 142514), anti‐FAS (BD, 554258), anti‐mouse CD38 (BD, 102721). The gating strategies of human Tfh cells, mouse T cell subsets and mouse B cell subsets were displayed in Figure S12.

*Chromatin isolation by RNA purification (ChIRP) assay*: The Magna ChIRP RNA interactome kit was carried out. Assay was conducted following the standard instruction. Overall, 1% glutaraldehyde was used to cross link the Tfh cells. The prepared samples were sheared by sonication. IL21‐AS1 probes were added into cell lysate for hybridization. The eluted proteins were determined by liquid chromatography‐tandem MS.

*Statistical analysis*: GraphPad Prism 8.0 and SPSS 19.0 were used to organize and analyse data. In order to compare two groups and multiple groups, unpaired or paired two‐tailed *t*‐test and one‐way analysis of variance were used respectively. *p < .05, **p < .01, ***p < .001.

## CONFLICTS OF INTEREST

The authors declare no competing interests.

## Supporting information

Supporting Information

Supporting Information

Supporting Information

Supporting Information

Supporting Information

Supporting Information

Supporting Information

Supporting Information

## Data Availability

The RNA‐seq profiles have been deposited in the National Omics Data Encyclopedia (NODE) with primary accession code OEP003549. All other remaining data supporting to conclusion of this study are available in article and supplementary files.
