# Supplementary material for: LncRNA IL21‐AS1 interacts with hnRNPU protein to promote IL21 overexpression and aberrant differentiation of Tfh cells in systemic lupus erythematosus
Source: Clin Transl Med. 2022 Nov 29;12(12):e1117. doi: 10.1002/ctm2.1117 (PMC9708910; doi:10.1002/ctm2.1117)
Supplement: Supplementary file 1 — Supporting Information [file CTM2-12-e1117-s005.docx]

**LncRNA IL21-AS1 interacts with hnRNPU protein to promote IL-21 overexpression and aberrant differentiation of Tfh cells in systemic lupus erythematosus**

**Supplementary figures**


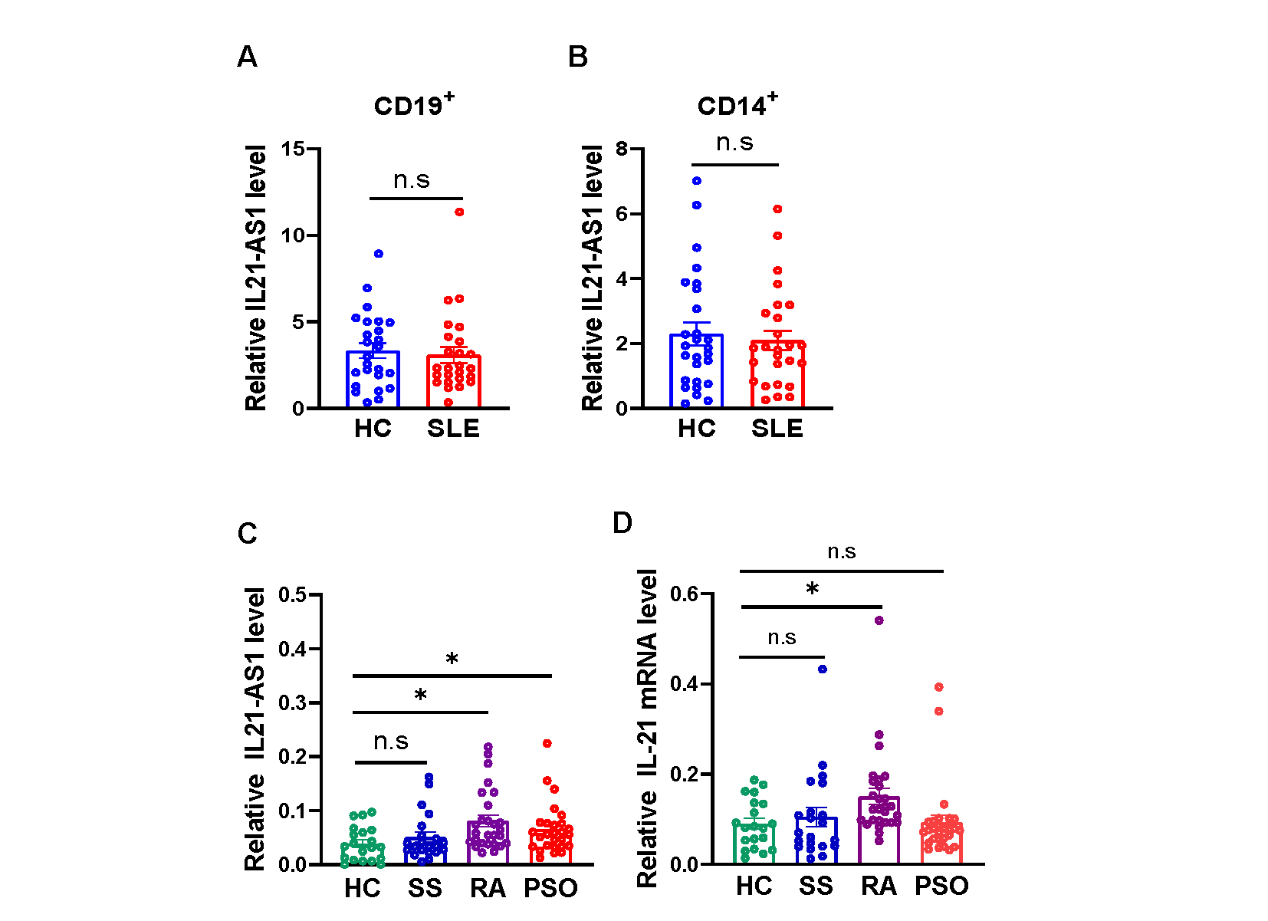


**FIGURE S1. The expression of IL21-AS1 in CD19^+^, CD14^+^ cells in SLE patients and CD4^+^ cells in SS, RA and PSO patients.**

**(A)** RT-qPCR detected the expression of IL21-AS1 in CD19^+^B cells of SLE patients (n=25) and healthy controls (n=25). **(B)** RT-qPCR detected the expression of IL21-AS1 in CD14^+^ monocyte of SLE patients (n=27) and healthy controls (n=27). **(C)** RT-qPCR detected the expression of IL21-AS1 in CD4^+^ cells of SS (n=21), RA (n=27), PSO (n=27) patients and healthy control (n=20). **(D)** RT-qPCR detected the expression of IL-21 mRNA in CD4^+^ cells of SS (n=21), RA (n=27), PSO (n=27) patients and healthy control (n=20). Data are shown as the means ± S.E.M. **P* < 0.05, ***P* < 0.01, ****P*< 0.001 relative to controls. *P*-value was determined using two-tailed student’s *t*-tests.


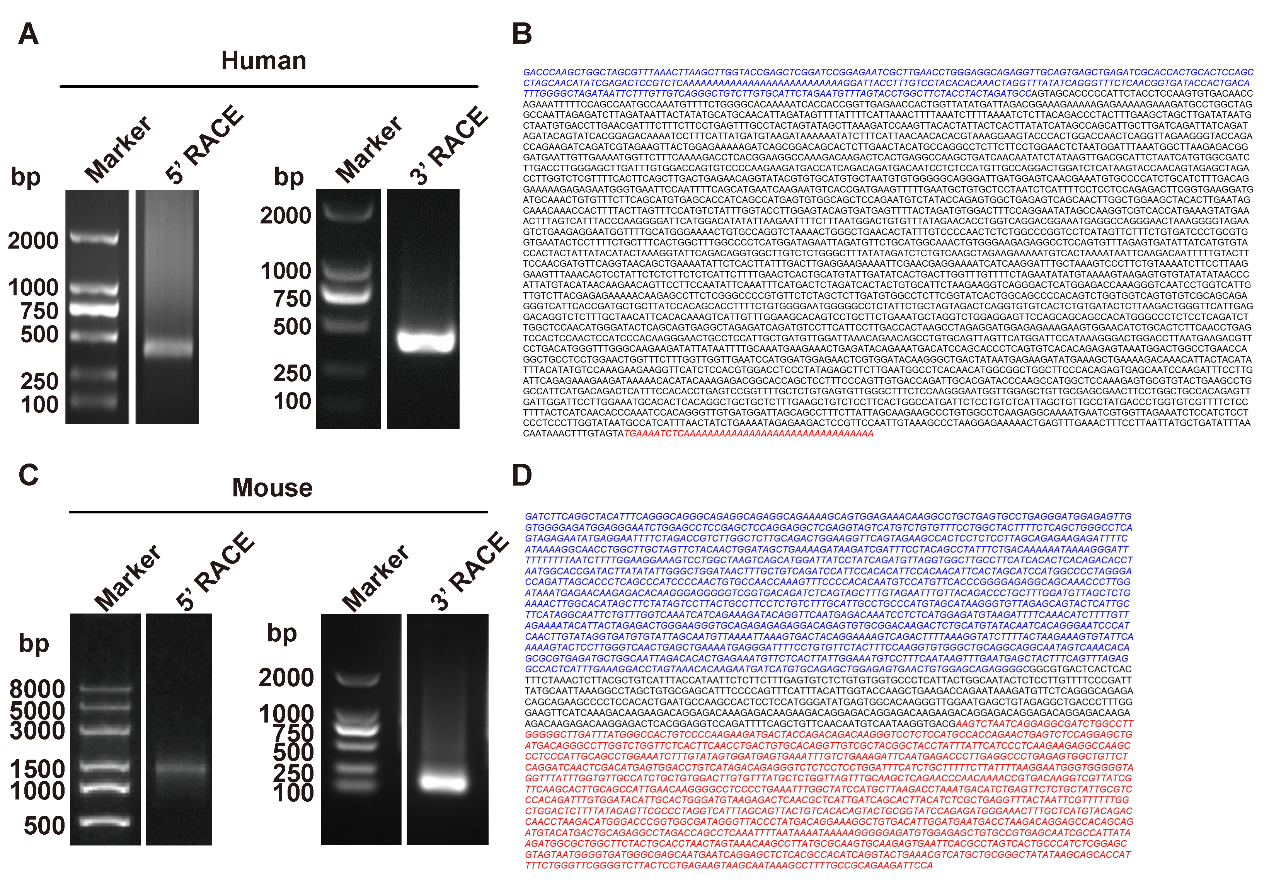


**FIGURE S2. Cloning and sequence of IL21-AS1 and mIl21-AS1 by 5' RACE and 3' RACE.**

**(A)** Agarose gel electrophoresis detected the length of product by 5' RACE and 3' RACE in human Tfh cells. **(B)** Full-length sequence of IL21-AS1, sequence in blue was amplified by 5' RACE, sequence in red was amplified by 3' RACE. **(C)** Agarose gel electrophoresis detected the length of product by 5' RACE and 3' RACE in mouse Tfh cells. **(D)** Full-length sequence of mIl21-AS1, sequence in blue was amplified by 5' RACE, sequence in red was amplified by 3' RACE.


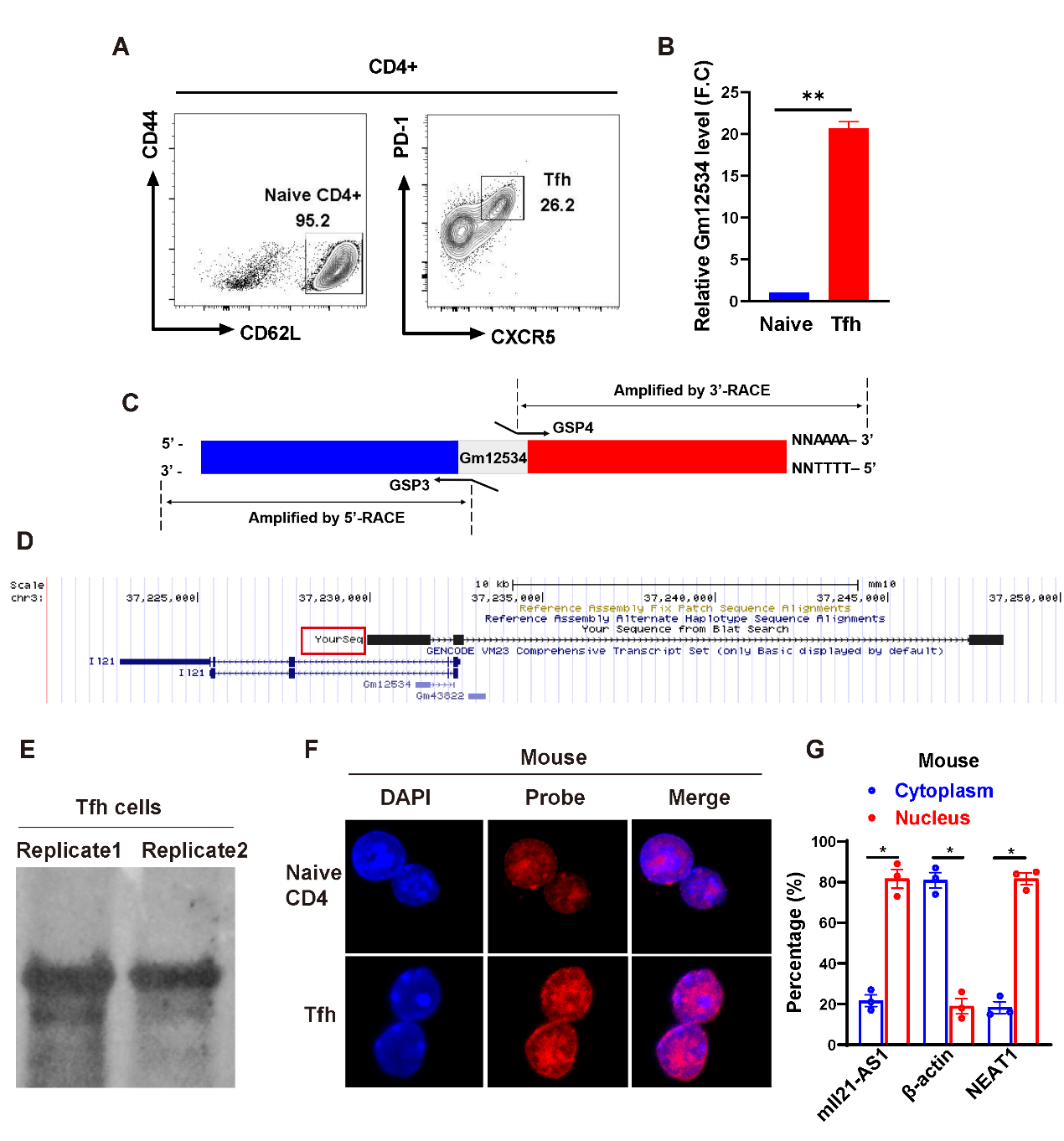


**FIGURE S3. Cloning and identification of mouse IL21-AS1.**

**(A)** Naïve CD4^+^ T cells (CD4^+^CD44^-^CD62L^+^) were isolated from WT mice and induced to Tfh cells (CD4^+^PD-1^+^CXCR5^+^) for 3 days under Tfh polarization condition. **(B)** RT-qPCR detected Gm12534 expression in naïve CD4^+^T cells and induced Tfh cells. **(C)** mIl21-AS1 RACE model. **(D)** Sequence blast showed that the location of mouse IL21-AS1 was at the 5'end of *Il21* gene in the genome, opposite to transcription direction of IL21 gene. **(E)** Northern blot detected mIl21-AS1 expression in mouse-Tfh5 cells. **(F)** expression and distribution of IL21-AS1 in mouse naïve CD4^+^T cells and Tfh cells by FISH. **(G)** RT-qPCR detected the percentage of mIl21-AS1 in nucleus and cytoplasm. Data are representative of three independent experiments (mean ± S.E.M, n=3). **P* < 0.05, ***P* < 0.01, ****P*< 0.001 relative to controls. *P*-value was determined using two-tailed student’s *t*-tests.


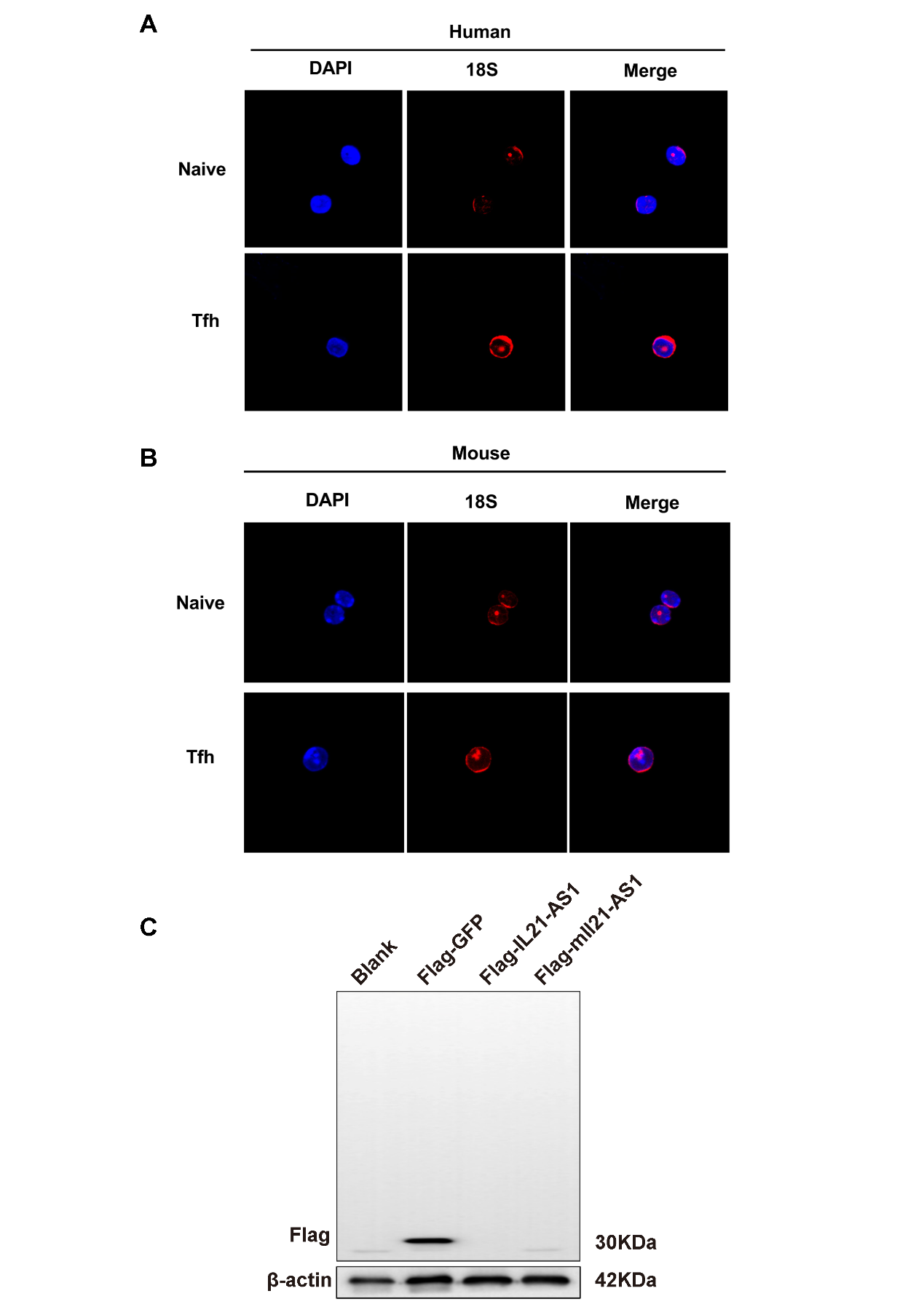


**FIGURE S4. 18S staining by FISH and coding ability detection of IL21-AS1 and mIl21-AS1 by western blot.**

**(A)** 18S staining by FISH as cytoplasmic-only control in human. **(B)** 18S staining by FISH as cytoplasmic-only control in mouse. **(C)** The sequences of IL21-AS1, mIL2-AS1 and GFP were cloned into eukaryotic expression vector with flag label. Western blot was used to detect the fusion protein expression.


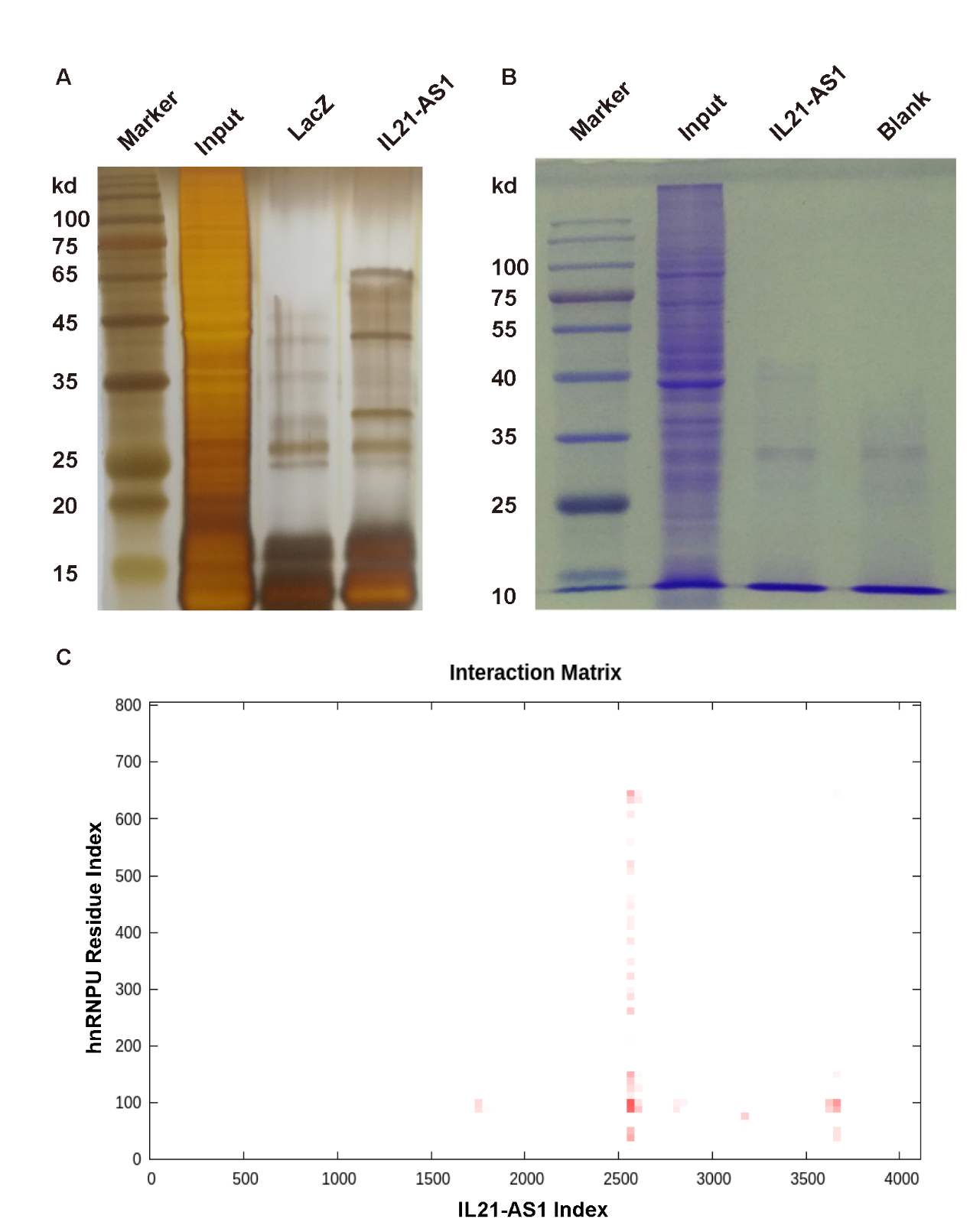


**FIGURE S5. ChIRP and RNA pulldown detected IL21-AS1 binding protein.**

**(A)** ChIRP with Mass Spectrometry (MS) detected binding protein of IL21-AS1 in human Tfh cells. The silver staining PAGE-SDS gel showed protein marker (lane 1), input (lane 2), LacZ probe control (lane 3), and IL21-AS1 probe (lane 4). **(B)** RNA pull-down with MS detected of IL21-AS1 binding protein in human Tfh cells. The coomassie brilliant blue staining PAGE-SDS gel showed protein marker (lane 1), input (lane 2), IL21-AS1 probe (lane 3), and blank control (lane 4). **(C)** Predicted protein binding regions in IL21-AS1 nucleotide sequence with hnRNAPU protein by CatRAPID software, Red indicates protein residue index. A potential protein binding is shown in the sequence 2543nt-2708nt.


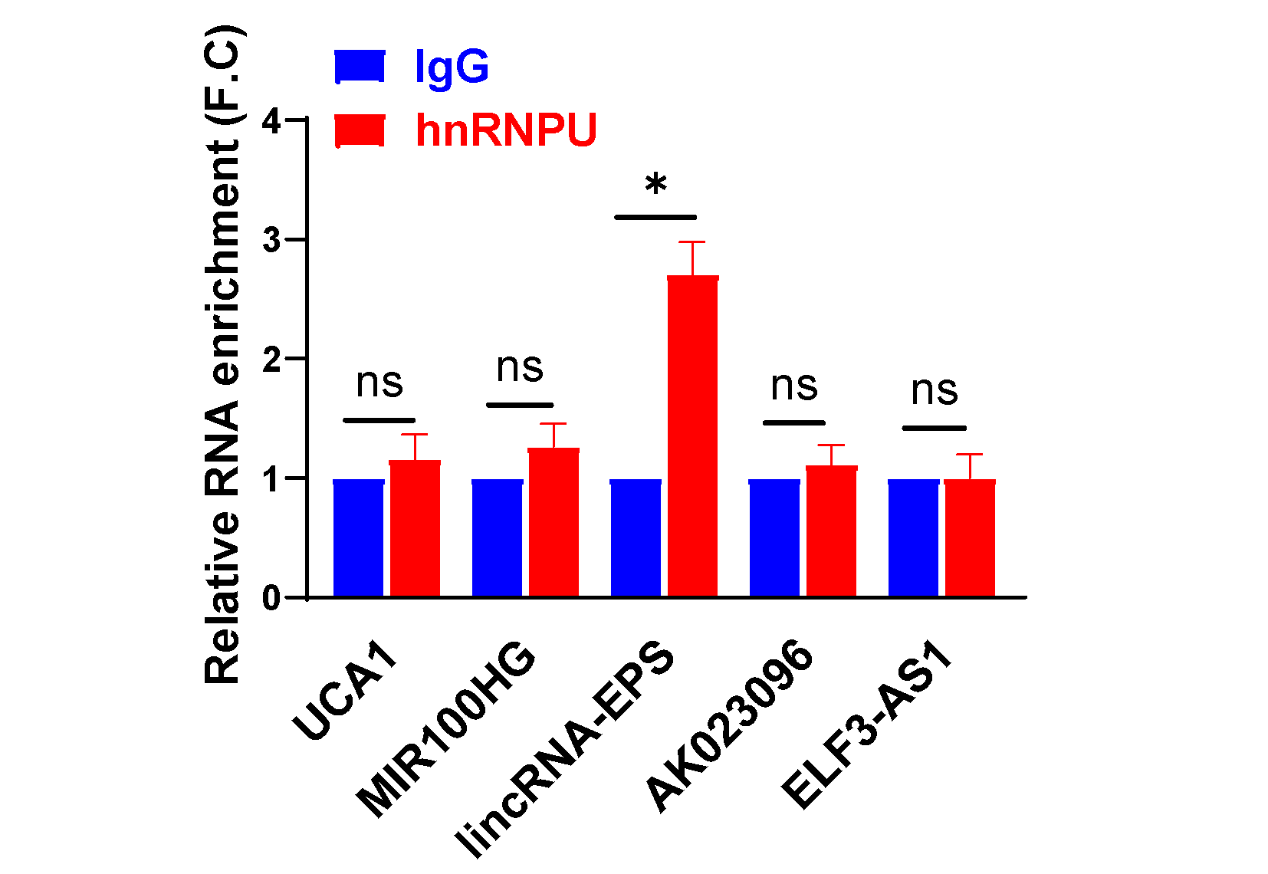


**FIGURE S6. hnRNPU binding of negative control RNA by RIP-qPCR.**

UCA1, MIR100HG, lincRNA-EPS, AK023096 and ELF3-AS1 were selected as negative control, RIP-qPCR analyzed their interaction with hnRNPU protein. **P* < 0.05 relative to controls. *P*-value was determined using two-tailed student’s *t*-tests.


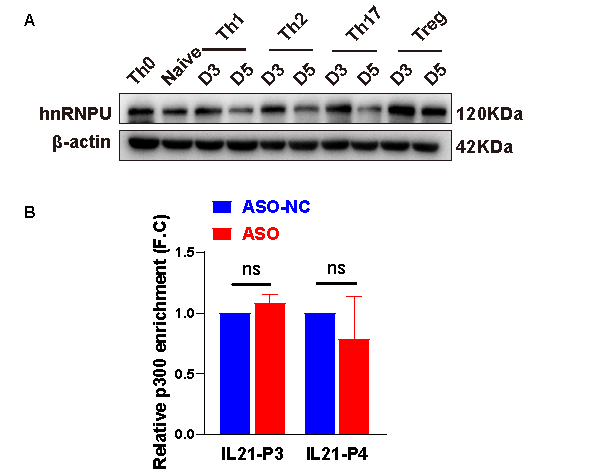


**FIGURE S7. HnRNPU expression in T cell subsets by western blot.**

**(A)**Naïve cells from healthy donors were isolated and induced into Th1/2/17/Treg under polarization condition, cells were harvest at day3 and day5 after induction, western blot was used to detect hnRNPU expression in induced T cells subsets. **(B)** ChIP-qPCR analyzed the enrichment level of P300 in the *IL21* gene proximal promoter region in IL21-AS1 ASO group and control group.


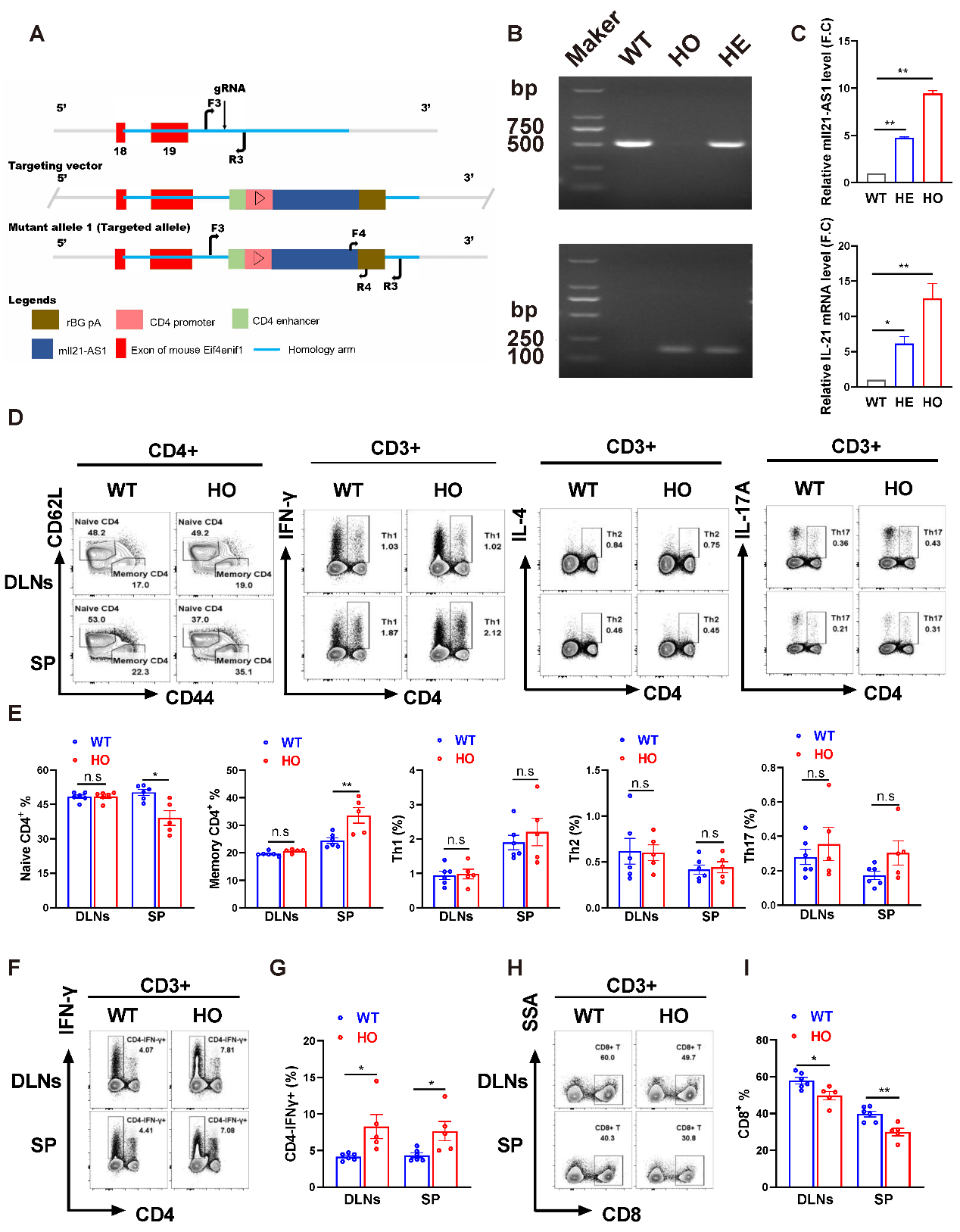


**FIGURE S8. Construction of mIl21-AS1 knock-in mice and T cells subsets in mice.**

**(A)** Targeting strategy of mIl21-AS1 knock-in mice. **(B)** PCR and agarose gel identified mouse genotypes for wild-type mice (WT), heterozygous (HE) and homozygous mice (HO). **(C)** Detection of mIl21-AS1 and IL-21 mRNA level in WT, HE and HO mice by RT-qPCR. **(D)** Representative flow diagram of naïve CD4^+^T cells (CD4^+^CD44^-^CD62L^+^), memory CD4^+^T cells (CD4^+^CD44^+^CD62L^-^), Th1 cells (CD4^+^IFNγ^+^), Th2 cells (CD4^+^IL-4^+^) and Th17 cells (CD4^+^IL-17A^+^) in DLNs and spleen of WT and HO mice. **(E)** Statistical analysis of percentages of naïve CD4^+^T cells, Memory CD4^+^T cells, Th1 cells, Th2 cells and Th17 cells in WT (n=6) and HO (n=5) mice. **(F)** Representative flow diagram of CD4^-^IFNγ^+^ cells in WT and HO mice. **(G)** Statistical analysis of percentage of CD4^-^IFNγ^+^ cells in WT (n=6) and HO (n=5) mice. **(H)** Representative flow diagram of CD8^+^T cells in WT and HO mice. **(I)** Statistical analysis of percentage of CD8^+^T cells in WT (n=6) and HO (n=5) mice. Data are shown as the means ± S.E.M. **P* < 0.05, ***P* < 0.01, ****P*< 0.001 relative to controls. *P*-value was determined using two-tailed student’s *t*-tests.


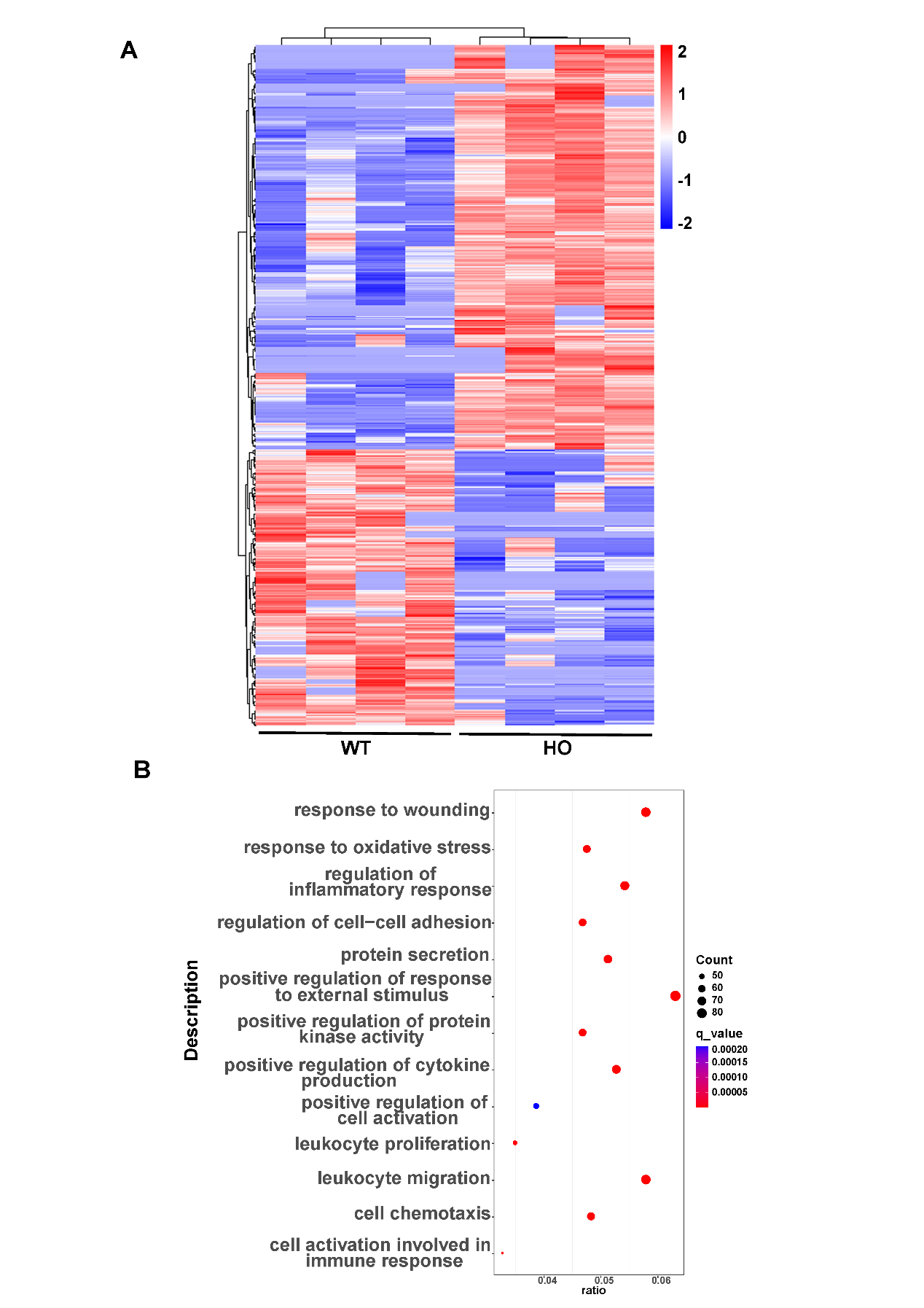


**FIGURE S9. cluster and GO analysis according to RNA-seq of CD4^+^T cells from WT and HO mice**

**(A)** heatmap showed up-regulated and down-regulated genes between CD4^+^T cells from WT(n=4) and HO mice(n=4). **(B)** GO analysis of up-regulated genes in CD4^+^T cells from HO mice.


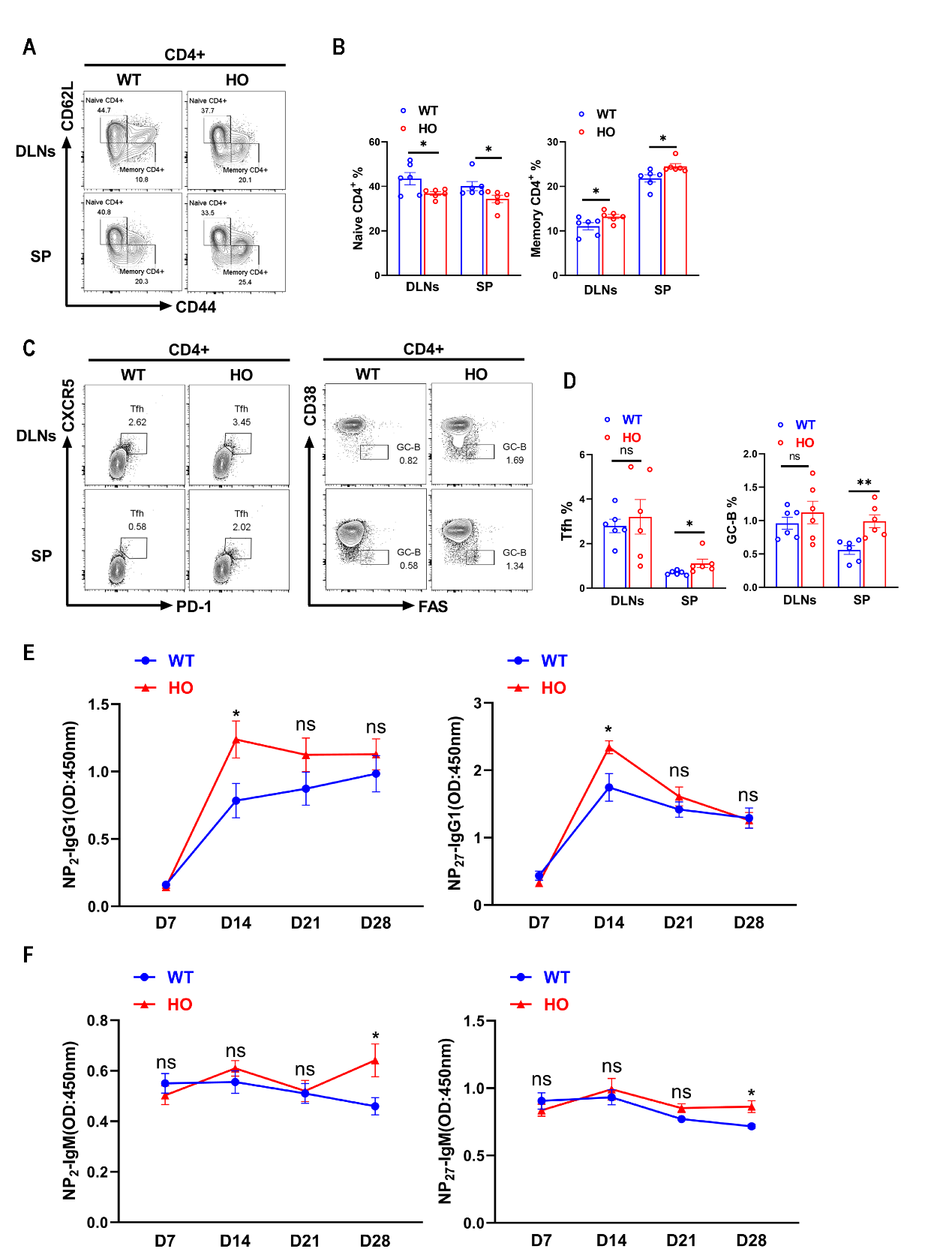


**FIGURE S10 Stimulation of HO and WT mice with NP-KLH**

**(A)** Representative flow diagram of naïve CD4^+^T and memory CD4^+^T. **(B)** Statistical analysis of percentages of naïve CD4^+^T and memory CD4^+^T in HO and WT mice immunized by NP-KLH (n=6). **(C)** Representative flow diagram of Tfh cells and GC-B cells. **(D)** Statistical analysis of percentages of Tfh cells and GC-B cell in HO and WT mice immunized by NP-KLH (n=6). **(E)** NP_2_- and NP_27_- specific IgG1 in HO and WT mice immunized by NP-KLH (n=6) at day7, day14, day21 and day28. **(F)** NP_2_- and NP_27_- specific IgM in HO and WT mice immunized by NP-KLH (n=6) at day7, day14, day21 and day28.


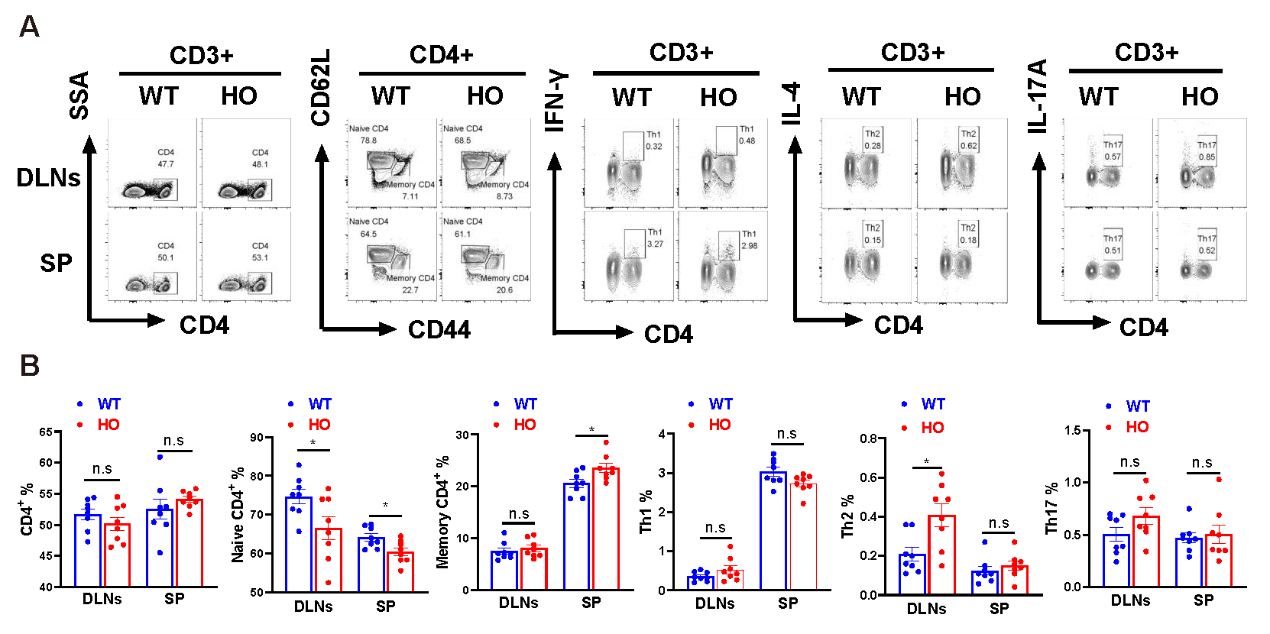


**FIGURE S11. Difference of naïve CD4^+^T, memory CD4^+^T and Th1/2/17 between WT and HO cells-induced lupus mice.**

**(A)** Representative flow diagram of naïve CD4^+^T, memory CD4^+^T and Th1/2/17 cells in cGVHD mice induced by WT-derived lymphocytes (n=8) and HO-derived lymphocytes (n=8). **(B)** Statistical analysis of percentages of naïve CD4^+^T, memory CD4^+^T and Th1/2/17 cells in cGVHD mice induced by WT-derived lymphocytes and HO-derived lymphocytes (n=8). Data are shown as the means ± S.E.M. **P* < 0.05, ***P* < 0.01, ****P*< 0.001 relative to controls. *P*-value was determined using two-tailed student’s *t*-tests.





**FIGURE S12. Gating strategy of flow cytometry**

**(A)** Gating strategy of human Tfh cells. **(B)** Gating strategy of mouse T cells subsets. **(C)** Gating strategy of mouse B cells subsets.

**Supplementary tables**

**Supplementary table S1.** The differentially expressed lncRNAs and genes in CD4^+^ T cells between SLE patients and healthy controls.cha

**Supplementary table S2.** The differentially expressed lncRNAs and genes between induced Tfh cells and naïve CD4^+^ T cells.

**Supplementary table S3.** Probes for ChIRP assay.

**Supplementary table S4.** The protein identified by ChIRP and Mass Spectrometry.

**Supplementary table S5.** The protein identified by RNA pulldown and Mass Spectrometry.

**Supplementary table S6.** Differentially expressed genes in CD4^+^T of HO mice compared with WT mice.

**Supplementary table S7.** Basic information of SLE patients and healthy subjects.

**Supplementary table S8.** Differentiation condition of human naïve CD4+T into T cell subsets and Activation condition of CD4^+^T cells

**Supplementary table S9.** Primer sequences of RT-qPCR and ChIP-qPCR

**Supplementary table S10.** 5’ and 3’RACE Specific amplification primers (GSP)

**Supplementary table S11.** The primers of northern blot
