# Supplementary material for: LncRNA IL21‐AS1 interacts with hnRNPU protein to promote IL21 overexpression and aberrant differentiation of Tfh cells in systemic lupus erythematosus
Source: Clin Transl Med. 2022 Nov 29;12(12):e1117. doi: 10.1002/ctm2.1117 (PMC9708910; doi:10.1002/ctm2.1117)
Supplement: Supplementary file 8 — Supporting Information [file CTM2-12-e1117-s007.docx]

**Supplementary Table S7.** Basic information of SLE patients and healthy subjects.

| SLE | Gender | Age | SLEDAI | NC | Gender | Age |
| --- | --- | --- | --- | --- | --- | --- |
| 1 | female | 33 | 16 | 1 | female | 33 |
| 2 | female | 38 | 18 | 2 | female | 38 |
| 3 | female | 24 | 4 | 3 | female | 26 |
| 4 | female | 20 | 10 | 4 | female | 28 |
| 5 | male | 52 | 6 | 5 | female | 32 |
| 6 | female | 19 | 8 | 6 | male | 19 |
| 7 | female | 27 | 12 | 7 | female | 22 |
| 8 | female | 28 | 3 | 8 | female | 22 |
| 9 | female | 50 | 19 | 9 | female | 32 |
| 10 | female | 27 | 12 | 10 | female | 27 |
| 11 | female | 54 | 10 | 11 | female | 24 |
| 12 | female | 28 | 25 | 12 | male | 28 |
| 13 | female | 30 | 28 | 13 | female | 26 |
| 14 | female | 38 | 16 | 14 | female | 38 |
| 15 | female | 32 | 2 | 15 | female | 31 |
| 16 | female | 57 | 6 | 16 | female | 47 |
| 17 | female | 26 | 26 | 17 | female | 26 |
| 18 | female | 38 | 2 | 18 | female | 32 |
| 19 | female | 18 | 12 | 19 | female | 18 |
| 20 | male | 48 | 12 | 20 | male | 28 |
| 21 | female | 36 | 2 | 21 | female | 37 |
| 22 | female | 25 | 4 | 22 | female | 25 |
| 23 | female | 52 | 4 | 23 | male | 42 |
| 24 | female | 25 | 10 | 24 | female | 25 |
| 25 | female | 48 | 13 | 25 | female | 39 |
| 26 | female | 29 | 6 | 26 | female | 22 |
| 27 | female | 26 | 12 | 27 | female | 26 |
| 28 | female | 40 | 1 | 28 | female | 55 |
| 29 | female | 34 | 11 | 29 | male | 34 |
| 30 | female | 25 | 5 | 30 | female | 25 |
| 31 | male | 64 | 6 | 31 | female | 54 |
| 32 | female | 20 | 6 | 32 | female | 20 |
| 33 | female | 56 | 2 | 33 | female | 51 |
| 34 | female | 32 | 8 | 34 | female | 32 |
| 35 | female | 12 | 12 | 35 | female | 34 |
| 36 | male | 46 | 12 | 36 | male | 25 |
| 37 | female | 44 | 8 | 37 | female | 41 |
| 38 | female | 30 | 4 | 38 | female | 29 |
| 39 | female | 41 | 12 | 39 | female | 26 |
| 40 | female | 37 | 12 | 40 | female | 40 |

|  | Anti-CD3 | Anti-CD28 | Anti-IFNγ | Anti-IL-4 | TGF-β | IL-2 | IL-4 | IL-6 | IL-12 | IL-1β | IL-23 | IL-21 |
| --- | --- | --- | --- | --- | --- | --- | --- | --- | --- | --- | --- | --- |
| Th0 | 5μg/ml | 2μg/ml | 10μg/ml | 10μg/ml |  |  |  |  |  |  |  |  |
| Treg | 5μg/ml | 2μg/ml |  |  | 5ng/ml | 10ng/ml |  |  |  |  |  |  |
| Th17 | 5μg/ml | 2μg/ml | 10μg/ml | 10μg/ml | 5ng/ml |  |  | 25ng/ml |  | 12.5ng/ml | 25ng/ml |  |
| Th1 | 5μg/ml | 2μg/ml |  | 10μg/ml |  | 5ng/ml |  |  | 10ng/ml |  |  |  |
| Th2 | 5μg/ml | 2μg/ml | 10μg/ml |  |  | 5ng/ml | 25ng/ml |  |  |  |  |  |
| Tfh | 5μg/ml | 2μg/ml |  |  | 5ng/ml |  |  | 20ng/ml | 10ng/ml |  |  | 20ng/ml |
| Human  CD4^+^T | 2μg/ml | 1μg/ml |  |  |  |  |  |  |  |  |  |  |
| Mouse  CD4^+^T | 5μg/ml | 2μg/ml |  |  |  |  |  |  |  |  |  |  |

**Supplementary table S8.** Differentiation condition of human naïve CD4^+^T into T cell subsets and activation condition of CD4^+^T cells

**Supplementary Table S9.** Primer sequences of RT-qPCR and ChIP-qPCR

| Gene | primer sequence (5′-3′) |
| --- | --- |
| IL21-AS1 | F: AACTACATGCCAGGCCTCTT |
|  | R: AGGTCAAGATCGCCACATGA |
| h-IL-21 | F: TGGTCCCTGAATTTCTGCCAG |
|  | R: TTAGTTGGGCCTTCTGAAAGCA |
| h-β-actin | F: CATGTACGTTGCTATCCAGGC |
|  | R: CTCCTTAATGTCACGCACGAT |
| mIl21-AS1 | F: CAATACTCTCCTTGTTTTCCCGAT |
|  | R: ATCGCCTCCTGATTAGACTTCGT |
| m-IL-21 | F: CGCCTCCTGATTAGACTTCG |
|  | R: AAAACAGGCAAAAGCTGCAT |
| m-β-actin | F: AAGTGTGACGTTGACATCCG |
|  | R: TCTGCATCCTGTCAGCAATG |
| MIR100HG | F: GTGGCAGAGTAAGGGATGGA |
|  | R: GGGGATGAACCATTGACAAC |
| lincRNA-EPS | F: CAGATGAGAGAAGTGCGCGG |
|  | R: TGGCCTGTTGTACCATGTGAT |
| AK023096 | F: ATCTTGCAGGCTTCATGCTT |
|  | R: GGCAGGCTTTTTCCTAGCTT |
| ELF3-AS1 | F: CCACCACCATCTTTCCGAGT |
|  | R: TACGGGTGACAGGCTACAAA |
| UCA1 | F: CTCTCCATTGGGTTCACCATTC |
|  | R: GCGGCAGGTCTTAAGAGATGAG |
| IL21-P1 | F: AGGATGTCTCAGGGAGGGAC |
|  | R: TGAGGGTGAAGGGGAGAACT |
| IL21-P2 | F: CACGATCAGCATGCAAACCTC |
|  | R: TCCTTGAAGACACTAACCACGG |
| IL21-P3 | F: TGGGTTAAAAGAGTCTCAGGGA |
|  | R: AGATCCAAAGCCCAGAAATGGT |
| IL21-P4 | F: ACAGGTAAGATGCCAGGGGA |
|  | R: AGTCAACGAAATGTGCCCCA |
| Gm12534 | F: GTGGCACAAGGGTTGGAATG |
|  | R: GTGTACCTGGACCTCCGTGA |

**Supplementary Table S10.** 5’ and 3’RACE Specific amplification primers (GSP)

| gene | sequence of primer (5’-3’) |
| --- | --- |
| IL21-AS1（GSP1） | CACCACCGGTTGAGAACCACT |
| IL21-AS1（GSP2） | GCCTATGACCCTGGTGTCGTTT |
| mIl21-AS1（GSP3） | CCAGCATCTCACGCGCTGTGTTTG |
| mIl21-AS1（GSP4） | CTCACGCCACATCAGGTACTGAAACG |

**Supplementary Table S11.** The primers of northern blot

| gene | sequence of primer (5’-3’) |
| --- | --- |
| IL21-AS1 | F: CTTTCCAACGATGTTCAGGTAA |
|  | R: AATAGAGGCCCCCATTCCC |
| mIl21-AS1 | F: GTGTATTCAAAAAGTACTCCTTGGG |
|  | R: AAACTGGGGAAATGCTCGC |
